# Persisting fetal clonotypes influence the structure and overlap of adult human T cell receptor repertoires

**DOI:** 10.1371/journal.pcbi.1005572

**Published:** 2017-07-06

**Authors:** Mikhail V. Pogorelyy, Yuval Elhanati, Quentin Marcou, Anastasiia L. Sycheva, Ekaterina A. Komech, Vadim I. Nazarov, Olga V. Britanova, Dmitriy M. Chudakov, Ilgar Z. Mamedov, Yury B. Lebedev, Thierry Mora, Aleksandra M. Walczak

**Affiliations:** 1 Shemyakin-Ovchinnikov Institute of Bioorganic Chemistry, Moscow, Russian Federation; 2 Laboratoire de physique théorique, CNRS, UPMC and École normale supérieure, Paris, France; 3 Pirogov Russian National Research Medical University, Moscow, Russian Federation; 4 Masaryk University, Central European Institute of Technology, Brno, Czech Republic; 5 Laboratoire de physique statistique, CNRS, UPMC and École normale supérieure, Paris, France; Fred Hutchinson Cancer Research Center, UNITED STATES

## Abstract

The diversity of T-cell receptors recognizing foreign pathogens is generated through a highly stochastic recombination process, making the independent production of the same sequence rare. Yet unrelated individuals do share receptors, which together constitute a “public” repertoire of abundant clonotypes. The TCR repertoire is initially formed prenatally, when the enzyme inserting random nucleotides is downregulated, producing a limited diversity subset. By statistically analyzing deep sequencing T-cell repertoire data from twins, unrelated individuals of various ages, and cord blood, we show that T-cell clones generated before birth persist and maintain high abundances in adult organisms for decades, slowly decaying with age. Our results suggest that large, low-diversity public clones are created during pre-natal life, and survive over long periods, providing the basis of the public repertoire.

## Author summary

The enormous diversity of T-cell receptor (TCR) molecules allows our adaptive immune system to recognize and fight infections. TCRs are formed through the stochastic rearrangement of DNA. By analysing human repertoire sequences of identical twins using a statistical model for TCR formation, we identified T-cells that were exchanged between twin embryos during pregnancy. We exploited the slightly different recombination statistics between fetal clonotypes and mature ones to track their relative fractions in adult T-cell repertoires of different ages. We showed that the decay of fetal clonotypes with age is extremely slow, spanning several decades. Our findings suggest that an important part of our adaptive immune system is formed before birth.

## Introduction

The adaptive immune system relies on the diversity of T-cell repertoires to protect us from many possible pathogenic threats. Each T-cell expresses on its surface many copies of a unique T-cell receptor (TCR), which engages with antigenic peptides—from self or foreign proteins—presented by other cells through their Major Histocompatibility Complex (MHC) molecules. The binding strength between the TCR and the peptide-MHC complex, which is typically weak for self peptides, and strong for some foreign peptides, is a major factor in determining the onset of an immune response. Since each TCR is only specific to a small fractions of the possible peptides, the body needs to maintain a very large diversity of TCRs to be able to recognize any possible foreign peptide from pathogens. Understanding how this diversity is generated, and how it develops and matures with age, is thus paramount to understanding adaptive immunity.

TCR diversity is produced by the V(D)J recombination machinery which generates the repertoire *de novo* in each individual. Repertoire diversity is encoded not only in the set of specific receptors expressed in a given individual, but also in their relative abundances—the number of T-cells expressing each unique TCR—which can differ by orders of magnitude. These differences are in part due to antigenic stimulation (infection, vaccination), implying that clones increase their sizes in response to common or recurring infections. Despite this great diversity, different individuals—regardless of their degree of relatedness—do express a subset of the exact same receptors, called the *public* repertoire [1]. This overlap is often interpreted as the convergence of individual repertoire evolutions in response to common antigenic challenges [2]. Indeed, some public TCRs are known to recognize common pathogens such as the cytomegalovirus (CMV) or the Epstein-Barr virus (EBV) [3]. However, this interpretation is challenged by the fact that these two properties—large differences in clone sizes and public repertoires—are also observed in naive repertoires, for which antigenic stimulation is not expected to be important [4, 5].

An alternative explanation for public clones, which does not invoke convergent repertoire evolution, is that both abundant and public receptors are more likely to be produced by rearrangement, and just occur by coincidence [1, 6]. This idea is backed by some compelling evidence. First, the amount of clonotype sharing between pairs of individuals can be accurately predicted in both naive and memory pools from statistical models of sequence generation [7]. Second, the likelihood that a clonotype sequence is shared by individuals has been reported to correlate with its abundance [6, 8]. However the origin of this correlation remains elusive. In addition, public clonotypes often have few or no randomly inserted N nucleotides, which limits their diversity [6]. Terminal deoxynucleotidyl transferase (TdT), the enzyme responsible for N insertions, is inactive in invariant T-cell subsets [9] and in some fetal T-cell clones. These subsets could contribute to the emergence of the public repertoire. Another confounding factor is the ageing of repertoires, and the concomitant loss of diversity, which is expected to affect the structure of clonal abundances as well as the repertoire’s sharing properties. How do all these effects shape the structure and diversity of TCR repertoires, and control their functional capabilities? Here we propose and test the hypothesis that a sizeable fraction of public clonotypes are created before birth. These clonotypes have low diversity because of reduced TdT activity, making them more likely to be shared among unrelated invididuals. Their large abundances, due to reduced homeostatic pressures in the early stages of repertoire development, allow them to survive over long periods.

## Results

### Clonotype sharing between individuals

We first examined in detail the question of clonotype sharing between individuals. Each TCR is a heterodimer made of two chains encoded by two distinct genes. Each gene is formed in the thymus by assembling together two or three gene templates from a finite set of germline segments—V and J segments for the *α* chain, and V, D and J segments for the *β* chain. In addition to the large diversity created by the combinatorial choice of germline segments, further diversity is produced by randomly deleting base pairs from the joining ends of the segments, and by inserting random non-templated (N) base-pairs at each junction. Each chain forms three loops, called Complementarity Determining Regions (CDR), which come in contact with the peptide-MHC complex during recognition. The first two loops, CDR1 and CDR2, are encoded in the germline V gene and are thought to interact mostly with the MHC. By contrast, the CDR3 concentrates most of the diversity, as it covers the junctions between the germline segments. The CDR3 interacts with the peptide directly, and is thus believed to play the biggest role in the recognition of foreign peptides.

After recombination, receptors are tested and selected for function and lack of auto-reactivity. The recombination mechanism frequently produces non-functional (also called nonproductive) receptor sequences, typically containing frameshifts or stop-codons. If the recombination result of the first chromosome is nonproductive, the second chromosome will recombine. In case this second recombination is successful, the cell will contain two recombined genes—one productive and one nonproductive. To avoid confounding effects due to convergent selection (both selection in thymus and clonal expansion in response to infection), we first focused on out-of-frame receptor sequences, which are nonproductive and hence must result from these first unsuccessful recombination events. Because the cells that contain them owe their selection and survival to the productive gene on the second chromosome, these out-of-frame sequences give us direct insight into the raw V(D)J recombination process [10, 11], free of clonal selection effects. The number of shared clonotypes between two sets of clonotypes, or clonesets, is approximately proportional to the product of the cloneset sizes [8, 11, 12]. We call the ratio of the two the normalized sharing number. In the regime of rare convergent recombination, this number is equal to the probability that two independent recombination events give the same sequence; it is thus independent of the cloneset sizes, and provides an appropriate measure of sharing for comparing different pairs of datasets with different sequencing depths. Under the assumption that sharing occurs by pure chance, only due to convergent recombination, this number can be predicted using data-driven generative probabilistic models of V(D)J recombination accounting for the frequencies of the assembled V, D, and J gene segments and the probabilities of insertions and deletions between them [7, 11, 13, 14]. We can estimate sharing either of the entire nucleotide chain (alpha or beta), or of the CDR3.

### Twins share more clonotypes than unrelated individuals

**Fig 1:**
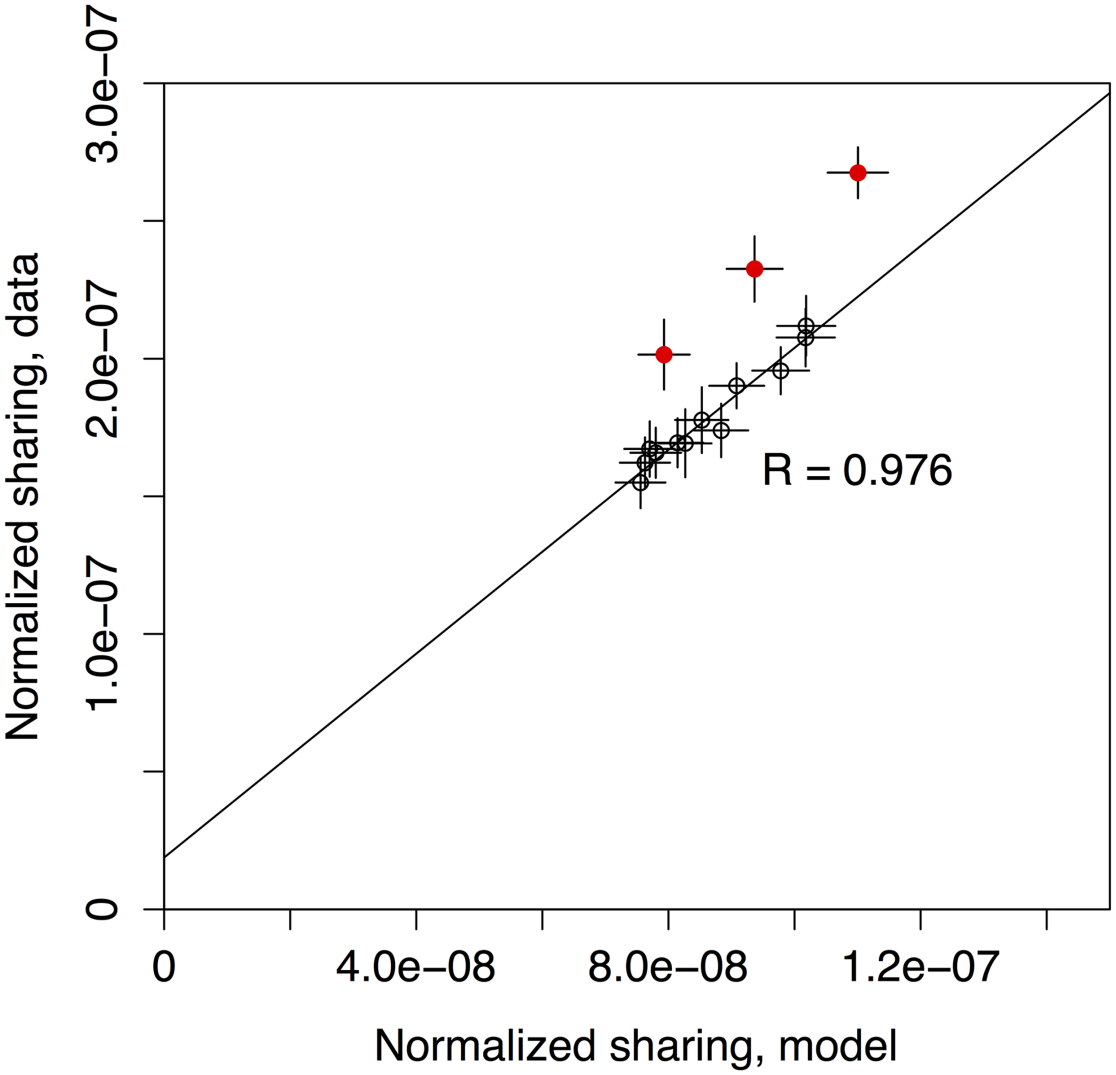
TCR out-of-frame repertoire sharing in monozygous twins is higher than in unrelated individuals, or than predicted by stochastic models of recombination. The number of shared out-of-frame alpha TCR clonotypes between all 15 pairs among 6 donors consisting of 3 twin pairs (ordinate) is compared to the model prediction (abscissa). To be able to compare pairs of datasets of different sizes, the sharing number was normalized by the product of the cloneset sizes. The three outstanding red circles represent the twin pairs, while the black circles refer to the 12 pairs of unrelated individuals among the 6 twins. The model prediction is based on a generative stochastic model of VJ recombination [13, 14], inferred separately for each donor to account for differences between individuals. It agrees well with the data from unrelated individuals up to a common multiplicative factor, but systematically underestimates sharing in twins. Error bars show one standard deviation.

Genetically identical individuals may be expected to have more similar recombination statistics due to similar recombination enzyme biases [8, 15–19], and therefore share more sequences. To assess these genetic effects, we looked at the sharing of TCR alpha and beta-chain receptor repertoires between three pairs of monozygous twins (6 individuals). We synthesized cDNA libraries of TCR alpha and beta chains from the donors’ peripheral blood mononuclear cells and sequenced them on the Illumina HiSeq platform (see S1 Fig and S1 Text). For each pair of individuals, the normalized number of shared out-of-frame alpha sequences was compared to the prediction from the recombination model trained on the out-of-frame repertoire of each individual, as shown in Fig 1 (see also S2 Fig for similar results on sharing of CDR3 nucleotide sequences). Sharing in unrelated individuals (the 12 non-twin pairs among 6 individuals, black circles) was well predicted by the model (Pearson’s *R* = 0.976), up to a constant multiplicative factor of 2.07, probably due to differences in effective cloneset sizes. While twins did share more sequences than unrelated individuals (the 3 twin pairs, red circles), this excess could not be explained by their recombination process being more similar. The model prediction was obtained by generating nucleotide sequences from models inferred using each individual’s cloneset as input [13, 14], mirroring their specific recombination statistics (see S1 Text). The normalized sharing number departed significantly from the model prediction only in twins, calling for another explanation than coincidence in that case. The same result was obtained for beta out-of-frame CDR3 nucleotide sequences (S3 Fig), although less markedly because of a lower signal-to-noise ratio due to smaller numbers of shared sequences. Most of beta out-of-frame nucleotide sequences shared among the highest-sharing twin pair associated with CD8 CD45RO+ (memory) phenotype in both individuals. This observation is surprising, because the non-functionality of these sequences excludes convergent selection as an explanation for it (see S1 Text for details).

We then examined the sharing of in-frame nucleotide CDR3 sequences. Most of in-frame sequences are functional, and have passed thymic and peripheral selection. Since these selection steps involve genetically-encoded HLA types (the type of MHC that cells express) and are therefore expected to be similar in related individuals, we wondered whether the functional repertoires of twins also displayed excess sharing. Remarkably, we found some excess sharing in the in-frame beta repertoire (S4 Fig), but none in the in-frame alpha repertoire (S5 Fig). However, the failure to observe excess sharing in this last case can be explained by the much higher expected number of shared nucleotide sequences in the alpha in-frame repertoire (due to both in-frame sequences being more numerous than out-of-frame ones, and to the lower diversity of alpha chains compared to beta chains) which could mask this excess in twins (see S1 Text).

### Low generation probabilities of excess shared clonotypes between twins suggest *in utero* T cell trafficking

**Fig 2:**
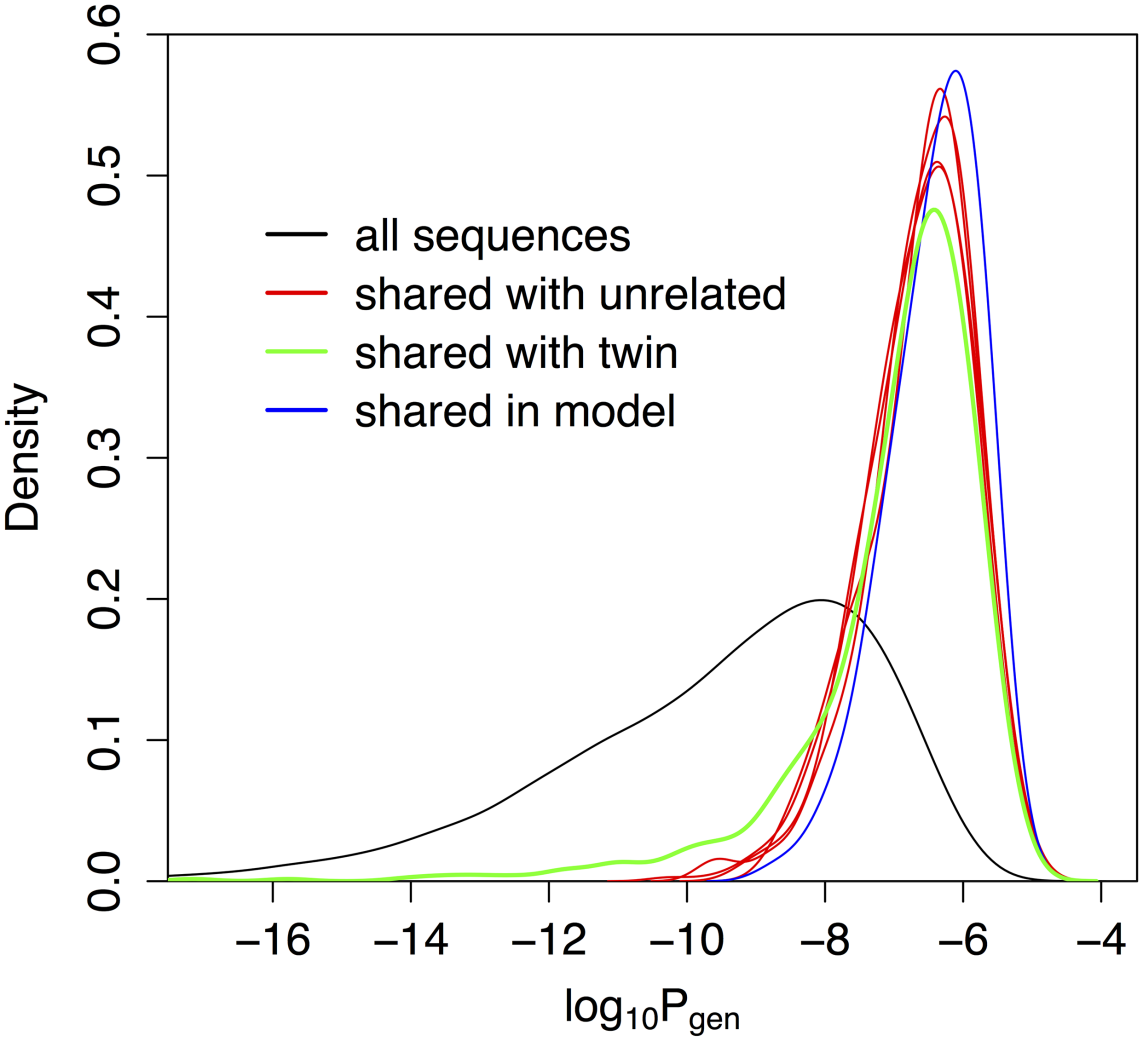
TCR nucleotide sequences shared between twins are statistically different from sequences shared between unrelated individuals. Distribution of log_10_ *P*_gen_, with *P*_gen_ the probability that a sequence is generated by the VJ recombination process, for shared out-of-frame TCR alpha clonotypes between one individual and the other five. While the distribution of shared sequences between unrelated individuals (red curves) is well explained by coincidental convergent recombination as predicted by our stochastic model (blue), sequences shared between two twins (green) have an excess of low probability sequences: 31 sequences with log_10_ *P*_gen_ < −10. For comparison the distribution of *P*_gen_ in regular (not necessarily shared) sequences is shown in black.

To investigate the origin of excess sharing between twins, we looked at the statistical properties of shared alpha out-of-frame nucleotide sequences from Fig 1. Shared clonotypes between non-twins, which happen by coincidence, should have a higher probability *P*_gen_ to have been produced by V(D)J rearrangement compared to non-shared clonotypes. Indeed, the distribution of *P*_gen_ among shared sequences, plotted in Fig 2, can be calculated from the probabilistic model of generation (blue curve), and the prediction agrees very well with the data between non-twins (red curves). By contrast, shared sequences between twins deviate from the prediction (green curve), especially in the tail of low-probability sequences, but are consistent with a mixture of 18 ± 3% of regular sequences (black curve), and the rest of coincidentally shared sequences (blue curve). These numbers agree well with the excess sharing in twins, which amounts to 17% ± 3% of non-coincidentally shared sequences, as estimated from Fig 1. Nucleotide sequences shared between twins also have higher numbers of insertions and are therefore longer than those shared between unrelated individuals or according to the model (S6 Fig, *p* = 2 ⋅ 10^−8^, two-sided t-test)—a trend that is even more pronounced in memory cells (S7 Fig, *p* < 10^−16^). Note these observations about recombination probabilities and the number of insertions are related: sequences with many insertions each have a low generation probability because of the multiplicity of inserted nucleotides.

Taken together, these observations support the existence of another source of shared sequences than coincidence in twins. Since the sharing of cord blood between twins is the only natural instance when the immune systems of two individuals share cells, we propose that the increased sharing of private TCRs between identical twins dates back to the sharing of cord blood cells, and that these shared clones persist into late age. This persistence of fetal clonotypes could be due to the long lifetime of the exchanged naive clones. Alternatively, long persistence could be achieved by the independent transition to memory of the shared clones in both twins.

### Sequences with no N insertions are enriched among abundant naive clonotypes in cord blood and in young adults

**Fig 3:**
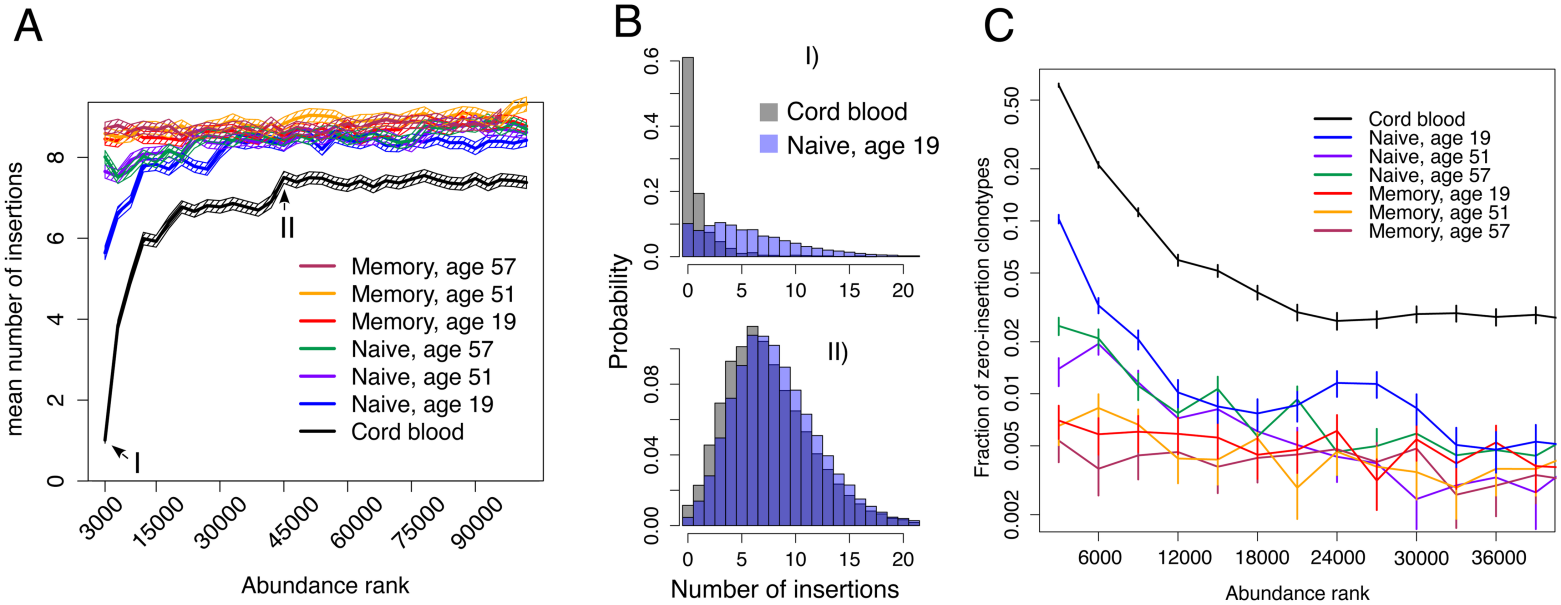
The number of inserted nucleotides in in-frame TCR beta clonotypes depends on their abundance. **A**. Mean numbers of insertions were obtained by analysing groups of 3000 sequences of decreasing abundance. Clonotypes from the cord blood (black) show a strong dependence on abundance, with high-abundance clones having much fewer insertions than low-abundance ones. Clonotypes in a young adult naive repertoire (blue) show a similar but less marked trend. Naive clonotypes in older adults (violet and green) show an even weaker trend. Adult memory samples of all ages show no dependence at all (red, yellow and maroon). Error bars show 2 standard errors. **B**. Probability distributions of the number of insertions in two rank classes, for young naive and cord-blood samples (ranks 1-3000 on top, ranks 45001-48000 on bottom). For high-ranking sequences, the probability of having zero insertions is high both for adult naive and cord blood samples. For middle-ranking sequences, the probability of 0 insertions is much lower, and the distributions are similar between adult naive and cord-blood samples. **C**. Fraction of clonotypes with zero insertions for different abundance classes. Error bars show one standard deviation. We present the analysis for independently published cord blood donors and different bin sizes in S11 and S10 Figs respectively.

To verify the hypothesis that clones formed during fetal life persist over long periods, we now turn to the analysis of data from unrelated individuals. We characterized the in-frame beta-chain repertoire of human cord blood and also three healthy non-twin adult donors of different ages (see Materials and methods and S1 Text). One feature of the rearranged chains is the number of insertions at the junctions between the gene segments (VD and DJ in the case of beta chains). We ranked beta TCR clonotypes from human cord blood data by decreasing abundances and plotted the mean number of insertions (inferred iteratively and averaged over groups of 3000 clonotypes, see S1 Text), as a function of this abundance rank (Fig 3A). The most abundant clones in cord blood had markedly smaller numbers of insertions (black line). The naive repertoire of a young adult (blue line) showed a much weaker dependence on abundance than the cord blood repertoire, but followed a similar trend. The dependence was even further reduced in older adults (purple and green lines). Interestingly, the number of insertions in the beta chains of the adult memory repertoire (red, orange and maroon lines) did not depend of the abundance of these cells. This observation can be explained by the resetting of the size of memory clones following an infection, erasing features of the abundance distribution inherited from fetal life. Looking more closely into the distribution of the number of insertions (Fig 3B) reveals that low mean numbers of insertions are associated with an enrichment in clonotypes with zero insertions. Accordingly, the fraction of naive zero-insertion sequences generally decreased with abundance rank (Fig 3C), with again a stronger dependency in cord blood and young adults. Fewer numbers of insertions in the cord blood are expected because TdT, the enzyme responsible for random insertions, is initially strongly downregulated in prenatal development [20, 21]. This enrichment in low-insertion sequences persists and shows weak signatures in the adult naive repertoire, suggesting long lifetimes of cord blood clonotypes (although not necessarily of individual cells).

### Abundant clonotypes with no N insertions decay slowly with age, but faster than the attrition of the naive cell pool

**Fig 4:**
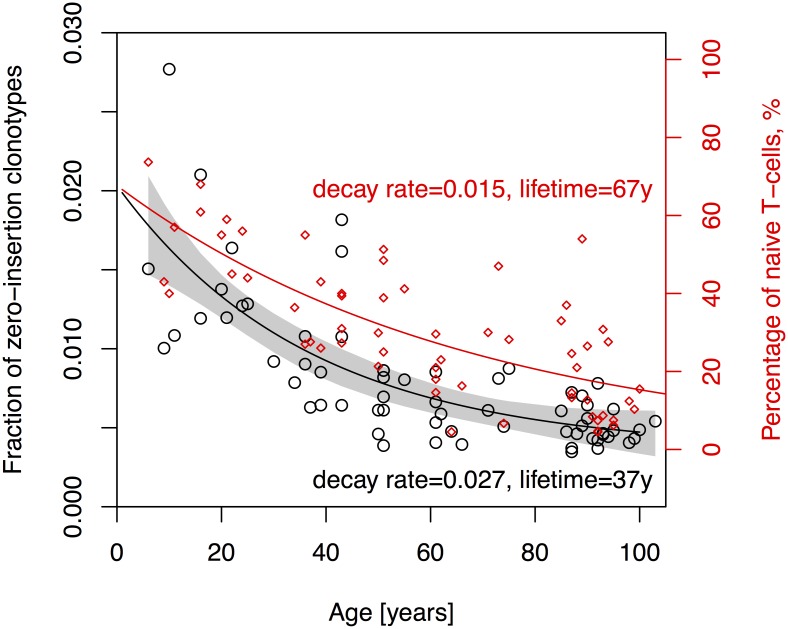
Lifetime of abundant in-frame TCR beta clonotypes with zero insertions. The fraction of zero-insertion clonotypes among the 2000 most abundant clonotypes in the unpartitioned repertoire as a function of age (black circles) is well fitted by an exponentially decaying function of time (black curve). This decay is faster than would be predicted from the decay of the naive compartment alone (red curve), indicating a slow decay of zero-insertion clonotypes of fetal origin. Red diamonds show percentage of naive T-cells measured using flow cytometry (see [23] for details). Scale of red axis was chosen so that the two decay curves start at the same point at age 0, and have the same long-time limit. We present the analysis for different bin sizes in S10 Fig.

The enrichment of zero-insertion sequences in large clonotypes of young people, relative to the baseline of zero-insertion clonotypes produced in adulthood, can be used to verify the hypothesis of long lived fetal clonotypes originating from the cord blood. Analysing publicly available TCR beta repertoire data from individuals of different ages [23, 24], we observed a slow decay of abundant zero-insertion clonotypes in the unpartitioned repertoire (memory plus naive) with age, with decay rate of 0.027 ± 0.009 yr^−1^, or a characteristic time of 37 years (Fig 4). However, the excess of abundant TdT- clonotypes of fetal origin only affects naive cells (Fig 3A), whose relative fraction in the repertoire is also known to decrease with time [23]. To assess the importance of this confounding effect, we fit an exponential decay model for the percentage of naive cells measured in same donors using flow cytometry (see S3 Table) and found a characteristic decay rate of 0.015 ± 0.002 yr^−1^, or a decay time of 67 years. The red curve in Fig 4, which shows the expected decay of zero-insertion clonotypes if it had been solely caused by the decay of the naive pool, does not agree with the data. Although the decay of naive cells within the top 2000 clonotypes could in principle be faster than in the overall T-cell population, we did not observed such an effect in the three individuals for which we have data partitioned into memory and naive clonotypes (see S1 Text I.G). Therefore, the attrition of the naive pool alone cannot explain the decrease of zero-insertion clonotypes, which we attribute instead to the progressive extinction of clones of fetal origin combined with their gradual replacement by newly generated naive cells. This is consistent with the hypothesis that excess clonotype sharing between twins is enabled by long-lived naive cells, but does not exclude the possibility that this excess sharing can be supported by memory cells as well.

### Clonotypes with zero N insertions quantitatively explain the relation between clonotype abundance and sharing between unrelated individuals

We have shown that abundant clones are enriched with zero-insertion sequences, both in the cord blood and in the adult naive repertoire. Zero-insertion clonotypes (regardless of their origin) are most likely to be shared by convergent recombination than regular sequences, because they are more likely to be generated due to reduced diversity. What are the implications of this observation for sharing between unrelated individuals? Since zero-insertion sequences are overrepresented among abundant clonotypes (Fig 3), we predict that abundant out-of-frame clones are more likely to be shared.

**Fig 5:**
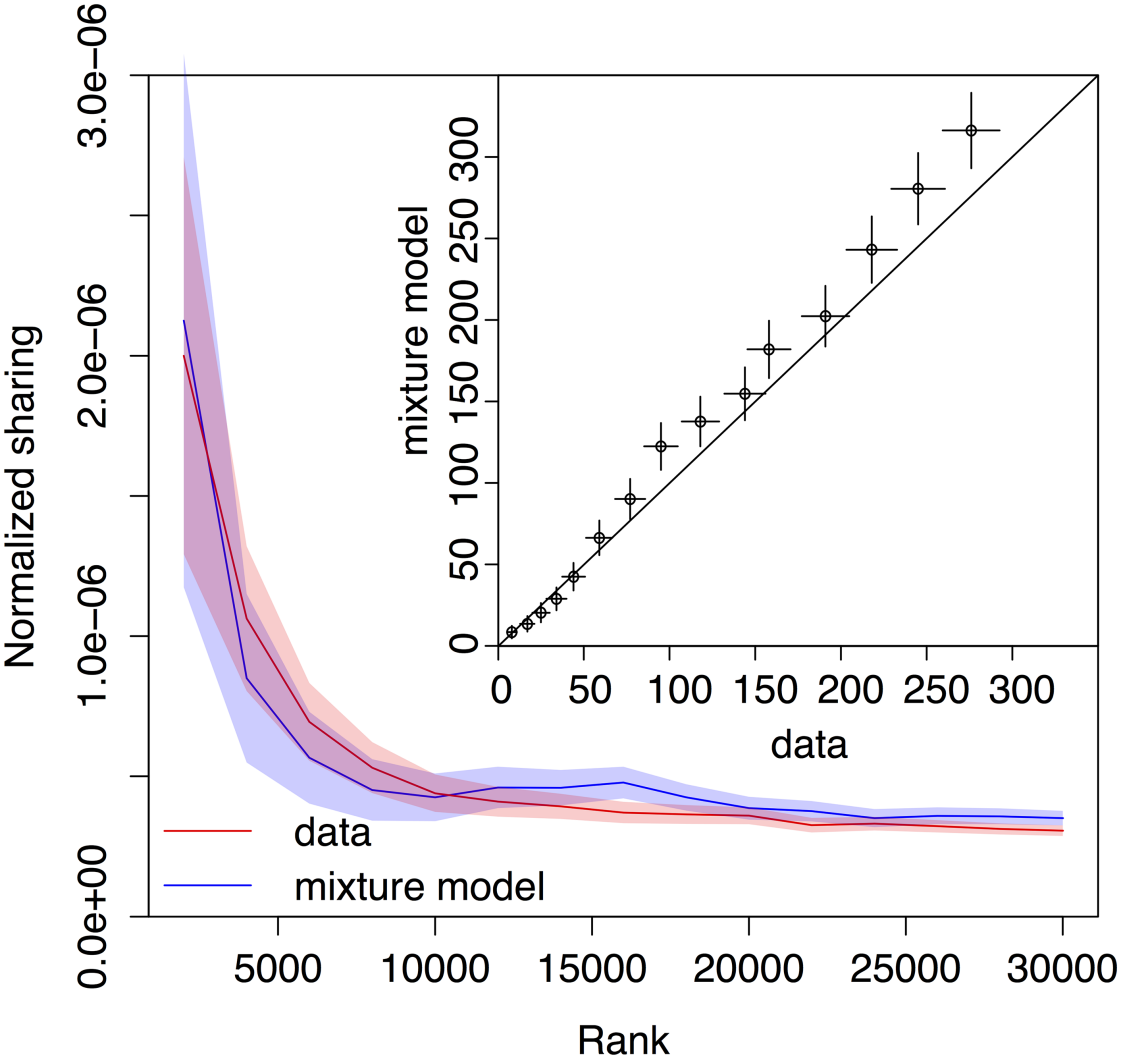
Sharing of alpha out-of-frame TCR clonotypes as a function of clonal abundance. The normalized number of shared out-of-frame alpha CDR3 nucleotide sequences between two individuals is showed as a function of clonotype abundance (e.g. normalized sharing for 2000 most abundant clones from both repertoires, 4000 most abundant, etc.), and compared to the amount of sharing that would be expected by chance (blue curve), taking into account the variable fraction of zero-insertion clonotypes as a function of their abundance. Data and predictions show excellent quantitative agreement (inset), with one fitting parameter. Error bars show one standard deviation.

To make our prediction quantitative, we built a mixture model of the out-of-frame alpha repertoire (see S1 Text for details). We assumed that clonotypes of a given abundance *C* are made up of a certain fraction *F*(*C*) of TdT-, zero-insertion clonotypes, and a complementary fraction 1 − *F*(*C*) of regular TdT+ clonotypes. Because TdT+ clonotypes may also have no insertions, the fraction of the TdT+ and TdT- sets had to be learned in a self-consistent manner. To learn these fractions, for each abundance class *C* we directly quantified the fraction *F*_0_(*C*) of sequences in the data that are consistent with zero insertions (i.e. can be entirely matched to the germline segments). Because non-templated nucleotides can coincide with the template, and also because TdT+ cells may have no insertions, *F*_0_(*C*) is not equal to *F*(*C*). However they are linearly related, so that it is enough for a model to agree with the data in terms of *F*_0_(*C*) to also guarantee agreement in terms of *F*(*C*). We generated a large number of nucleotide alpha out-of-frame sequences using our recombination model, and separated them into two groups: those that are consistent with no insertions (group A), and the others (group B). For each abundance class *C*, we created articifical datasets made of a fraction *F*_0_(*C*) of sequences from group A, and a fraction 1 − *F*_0_(*C*) from group B, where we recall that *F*_0_(*C*) is estimated from the data. We then repeated the sharing analysis in these artificial datasets in the same way as in the real datasets. The model accurately predicts the normalized sharing number of out-of-frame alpha-chain CDR3s as a function of clonotype abundance (Fig 5), up to the common multiplicative factor of 1.7 by which the non-mixture model generally underestimates CDR3 sharing (see S2 Fig). Thus, the enhanced sharing of high-abundance clonotypes is entirely attributable to their higher propensity to have no insertions, making them more likely to be shared by chance.

## Discussion

We found that adult twins present an interesting case of microchimerism in the adaptive immune system: shared rare TCR variants that recombined before birth survive for decades in their repertoires. We have also shown that adult naive repertoires, but not memory repertoires, have similar zero-insertion TCR clones distributions as cord blood repertoires. With age, the clone size distribution of naive adult repertoire becomes more similar to that of the memory repertoire. We hypothesize that this similarity between adult naive and cord blood repertoires is maintained by long lived fetal clones. Our results on the biological trafficking of T cells in twins are robust to possible experimental artefacts. First, our framework relies on the accurate counting of TCR cDNA sequences using unique molecular identifiers [25]. To exclude the possibility of contamination during the PCR and sequencing process, we double barcoded each cDNA library. To further exclude the possibility of early contamination of the blood samples, we performed replicate experiments at different times using different library preparation protocols. Comparison of repertoire overlaps from such replicate experiments for the same set of twins shows no difference and rules out experimental contamination as a confounding effect (see S1 Text). We also observed the same effects in previously and independently collected datasets [8], further excluding the possibility of experimental artefacts (S8 Fig). This reproducibility also suggests that the majority of out-of-frame sequences are not sequencing errors. Additional evidence for this fact comes from the different fractions of out-of-frame sequences observed in alpha and beta chains in TCR cDNA sequencing data, 13 and 3 percents respectively [8]– both of these fractions are much higher than the indel rate for the illumina platform [26, 27]. Our conclusions rely on a variety of data sources, and make extensive use of statistical analysis. As it is not yet possible to collect data from the same donors over many years, statistical evidence such as the amount of sharing in twins, or the amount of zero-insertion clonotypes versus age, is needed to investigate the evolution of repertoires over decades.

Cord blood sharing between twin embryos could have important implications on twin immunity: they could share and respond with private clonotypes, which would otherwise not be likely to be produced independently. This could possibly include sharing of malignant [28–30] or autoimmune clones, leading to disease in both individuals. In very rare cases such transfusion could also occur between dizigotic twins, leading to chimerism [31]. Anastomoses between monochorionic twin placentas are very common (more than 85 percent of uncomplicated pregnancies [32]), however the amount of exchanged blood may vary, and in some extreme cases it even leads to adverse outcomes such as twin-to-twin transfusion syndrome [33]. These effects could possibly affect the initial number of *in utero* shared clonotypes. This mechanism of sequence sharing is very different from sharing by convergent recombination [6], because it also implies the sharing of the second TCR chain and of the cell phenotype. Paired repertoires studies, which combine alpha and beta chains together [34, 35], could be used to track clones shared between twins more precisely, and distinguish them from convergently recombined ones.

Our results suggest two mechanisms with opposite effects on the sharing of clonotypes in twins as a function of the number of insertions. On the one hand, we have argued in Figs 1 and 2 that clonotypes shared through direct cell exchange should have a ‘normal’ number of insertions, because they are not due to random convergent recombination (which favors low numbers of insertions). On the other hand, we have shown in Fig 3 that cord blood cells are enriched in zero-insertion clonotypes, suggesting that clones shared in utero should be enriched in clonotypes with no of few insertions. Which one of these two effects dominate? TdT is suppressed in human embryos mostly in the first trimester of pregnancy [21]. Since TdT is active in the later trimesters the majority of the cord blood repertoire consists of clones with non-zero insertion numbers [22] similarly to the regular TdT+ post-natal clones. We show that the insertion distribution for non-abundant clones in cord blood closely resembles the insertion distribution observed in adults, with most clonotypes having insertions (see Fig 3B II). Such clonotypes could be exchanged in utero between twins, and easily identified as shared clonotypes with low *P*_gen_. Our theory predicts that twins should also exchange zero-insertion clonotypes, which are abundant in cord blood. However these shared clonotypes are indistinguishable from clonotypes shared by convergent recombination, which are also likely to have zero insertions. Therefore, the higher abundance of zero-insertion clonotypes in cord blood relative to mature repertoires does not contradict the observed sharing of high-insertion clonotypes due to cord blood exchange.

We have also showed that some of the clonotypes transferred in utero have the CD45RO+ phenotype, typical of central memory cells. It is possible that the longevity of these clones is connected with their memory status acquired early in life. To test this hypothesis, one would need to perform deep sequencing of purely sorted naive T-cells from adult twins and repeat the analysis presented in this paper. The transition from naïve to memory is also associated with clonal expansion, so it is possible that, within the *in utero* transfer hypothesis, the most easily detectable clonotypes shared between twins come from the memory population simply due to sampling effects. At the same time, the results plotted in Fig 3 suggest that naïve clonotypes may also be long lived. Thus, clonotypes transferred in utero in twins could be either of naive or memory origin.

Our conclusion that fetal clonotypes are long-lived is based on the analysis of over-abundant zero-insertion clonotypes. Invariant T-cells, MAIT (Mucosal-Associated Invariant T-cells) and iNKT (Invariant Natural Killer T-cells) are intrinsically insertion-less, have restricted VJ usage for alpha chain, and are often abundant. These cells are produced in adulthood and could in principle constitute a substantial fraction of our zero-insertion dataset, confounding our analysis. Since our abundant zero-insertion clonotypes have a very diverse usage of VJ genes, we can exclude that the majority of them are from invariant T-cells, although we did identify a small number of such invariant TCR alpha chain clonotypes, see S1 Text. An alternative explanation of the skewed zero-insertion clone size distribution of naive repertoires (see Fig 3A) is the existence of previously unknown subset of insertionless T-cells characterized by large proliferation activity, which would be produced in adulthood and make up the most abundant clones of the naive repertoire. To support this hypothesis, one would need to further assume that the production of these cells decays with age, to be consistent with the observations of Fig 4. Another related possibility is that insertionless clonotypes are generally favored by thymic selection, again in a age-dependent manner. However, in-frame clonotypes have been reported to be only moderately enriched (by less than 20%) in zero-insertion sequences relative to out-of-frame sequences (see Ref. [7], Fig 3E and 3F), meaning that thymic selection does not substantially favor zero-insertion clonotypes on average.

Our current data clearly shows that clonotypes that originated in the cord blood tend to be among the most abundant in the naive repertoire, but we cannot unambiguously point to the source of this effect. One possibility is convergent recombination [6, 36]: high clonotypes abundances could be due to the accumulation of multiple convergent recombination events made more likely by the limited recombination diversity during fetal development. However, we observed clonotypes with low generative probabilities among the most abundant clones in the cord blood repertoire, and also clonotypes with high generation probability among the least abundant clones. We conclude that convergent recombination alone could not predict cord blood clone frequencies. An alternative explanation is that these clones have had more time to expand than others. Fetal cells come from different precursors, and mature in a different environment (the fetal liver), than post-natal cells [37]. *In vitro* experiments have shown that fetal T-cells have a different proliferation potential than post-fetal cells [38]. Additionally, a vacant ecological niche effect may play a role. When these clones first appeared, the repertoire had not reached its carrying capacity set by homeostatic regulation, leaving room for future expansion. These clones may have initially filled the repertoire, later to be gradually replaced by post-fetal clonotypes. Consequently, fetal clones, including those whose TCR was recombined with no TdT, would be expected to have larger sizes. Quantitative TCR repertoire profiling (preferably with the use of unique molecular identifiers for accurate data normalization and error correction), performed for species with no TdT activity in the embryo, such as mice, as well as novel cell lineage tracking techniques [39] could be used to investigate the detailed dynamics of fetal clones. This large initial expansion of fetal clones could protect them from later extinction. This would suggest that the estimated 37-year lifetime of zero-insertion fetal clonotypes could be longer than that of regular clones produced after birth.

Sharing of beta TCRs has previously been shown to decrease with age [23]. Depletion of fetal clonotypes, which are more likely to be shared, could contribute to this phenomenon. Our results also predict that the excess sharing of clonotypes between twins due to the trafficking of fetal cells should decrease with age. In general, the observed abundance of large zero-insertion clonotypes and their persistence through significant part of our life should have important consequences for the adaptive immunity regulation both in pre- and post-fetal period. Interestingly, transgenic mice with induced fetal TdT expression showed impaired antibody response to certain bacterial pathogens, suggesting an important functional role of the low-diversity fetal repertoire in immune competence [20]. We could speculate that the primary target of these cells might be common pathogens with a long history of coevolution with humans, such as CMV and EBV.

Lastly, our general framework for analyzing the overlap between different repertoires has far-reaching practical implications for the tracking of T-cell clonotypes in the clinic. In particular, the analysis of overlap between pre- and post-treatment repertoires using probabilistic characteristics of clonotypes sharing could help determine the host or donor origin of clonotypes after hematopoietic stem cell transplantation (HSCT), and also increase reliability of malignant clones identification in minimal residual disease follow-up.

## Materials and methods

For a more detailed description of experimental and data analysis procedures see S1 Text Materials and Methods.

### NGS library preparation

RNA was isolated from the PBMC of healthy Caucasian donors: 3 pairs of female monozygotic twins (aged 23, 23 and 25 years old), 19 year old and 57 year old males, a 51 year old female and cord blood from a female newborn. CD4+ and CD8+ populations were isolated using CD4+ and CD8+ T-cell positive isolation kits (Invitrogen), CD45RO+ and naive cells were isolated from PBMC using CD45RO+ enrichment and human naive T-cell isolation kits (Myltenyi) respectively. cDNA of TCR alpha and beta chain was synthesized and sequenced on the Illumina HiSeq platform (see S1 Fig for library preparation technique, S1 Table for the oligonucleotides used, S2 Table for all samples and numbers of sequencing reads).

### Raw data processing

Raw data processing and data analysis were performed using published open-source software tools: MiGEC (https://github.com/mikessh/migec), MiXCR (https://github.com/milaboratory/mixcr/), tcR (https://github.com/imminfo/tcr) and repgenHHM (https://bitbucket.org/yuvalel/repgenhmm/downloads). We processed raw sequencing data with MiGEC [40] to extract unique molecular identifiers and we used MiXCR [41] to determine the CDR3 position. All raw data is available online on our server (see S1 Text Methods E. for the links) and also in Short Read Archive (SRP078490).

### Data analysis

Recombination models for beta and alpha chains were inferred using an EM-algorithm as described in [11, 13, 14], using the repgenHHM [13] and IGoR [14] software tools, selection models were inferred as described in [7]. The shared clonotype analysis was performed using the tcR package [42] and R statistical programming language [43]. To predict the number of shared out-of-frame clonotypes we generated random sequences using the recombination model parameters inferred separately for each individual in the previous step. We then filtered out-of-frame clonotypes and calculated the number of shared sequences between these simulated datasets using the tcR package.

To predict the number of shared in-frame clonotypes we also generated random sequences with recombination model parameters, filtered in-frame sequences and calculated the Q selection factors for each CDR3 amino acid sequence using selection models inferred separately for each individual. The number of shared sequences in the simulated in-frame datasets was reweighted by the Q factors as:

$$\begin{array}{c} \frac{1}{|S_{1}\left|\cdot \right|S_{2}|}\sum_{s\in S_{1}\cap S_{2}}Q^{\left(1\right)}\left(s\right)Q^{\left(2\right)}\left(s\right), \end{array}$$ (1)

where *S*_1_, and *S*_2_ are two synthetic sequence samples drawn from two models $P_{\text{gen}}^{\left(1\right)}$,$P_{\text{gen}}^{\left(2\right)}$ learned separately from the out-of-frame sequences of the two individuals, and *Q*^(1)^(*s*), *Q*^(2)^(*s*) are selection factors learned separately from these two individuals’ in-frame sequences. |*S*_1_| and |*S*_2_| denote the size of the two samples. The sum runs over sequences *s* found in both samples.

To estimate the distribution of the number of inserted nucleotides for different subsets of the repertoire (Figs 3 and 4), we used the same EM-algorithm when inferring the full repertoire models. To minimize the noise due to small subset sizes, we only learned the insertion distribution and took all other model parameters to be the same as in the previously inferred model in [11].

To fit the exponent decay of the ageing data we used the nlm2 R package. The data used in these fits is given in S3 Table. Fitting an exponentially decaying curve to the fraction *Z* of zero-insertion clonotypes in the 2000 most abundant clones as a function of age *T* (Fig 4):

$$\begin{array}{c} Z\approx c+a\exp(-bT), \end{array}$$ (2)

we found *c* = 0.00363 ± 0.00154, *b* = 0.0272 ± 0.0091 yr^−1^, and *a* = 0.016696± = 0.00188.

Fitting an analogous model for the attrition of the naive T-cell pool, *i.e.* the fraction *N* of naive T-cells as identified using flow cytometry (see [23] for details),

$$\begin{array}{c} N\approx a^{\prime }\exp\left(-b^{\prime }T\right). \end{array}$$ (3)

we obtained *a*′ = 0.68 ± 0.054 and *b*′ = 0.01485 ± 0.0018 yr^−1^.

### Ethics statement

All blood samples were taken in authorised diagnostics lab. All donors signed informed consent document for scientific use of their blood and publishing the results. Study was approved by local ethics committee and conducted in accordance with the Declaration of Helsinki.

## Supporting information

S1 Text Detailed description of experimental and data analysis procedures and supplementary results. (PDF)

S1 Fig Library preparation protocol. A) cDNA first strand synthesis for alpha and beta chains starts from specific primers in the C-segment conserved region. B) The template switching effect was used to introduce a universal primer binding site to the 3’cDNA end. The SMART-Mk sequence contains a sample barcode (black ellipse) for contamination control. C) and D) In two subsequent PCR steps we introduce the TruSeq adapter sequences along with Illumina sample barcodes (black ellipse). E) The resulting cDNA molecule is double barcoded, contains a Unique Molecular Identifier (UMI) and is suitable for direct sequencing on the Illumina HiSeq platform with the custom primers.
(PDF)

S2 Fig Number of shared out-of-frame alpha TCR CDR3 clonotypes reported between all 15 pairs of 6 donors consisting of 3 twin pairs (ordinate) compared to the model prediction (abscissa). To be able to compare datasets of different sizes, the sharing number was normalized by the product of the two cloneset sizes. The outlying three red circles represent the twin pairs, while the black circles refer to pairs of unrelated individuals. Error bars show one standard deviation. The diagonal line is a linear fit for unrelated individuals, of slope 1.7.
(PDF)

S3 Fig Number of shared out-frame beta TCR CDR3 clonotypes reported between all 15 pairs of 6 donors consisting of 3 twin pairs (ordinate) compared to the model prediction (abscissa). The three outlying red circles represent the twin pairs, while the black circles refer to pairs of unrelated individuals. Error bars show one standard deviation.
(PDF)

S4 Fig Number of shared in-frame beta TCR CDR3 clonotypes reported between all 15 pairs of 6 donors consisting of 3 twin pairs (ordinate) compared to the model prediction (abscissa). To be able to compare datasets of different sizes, the sharing number was normalized by the product of the two cloneset sizes. The three outlying red circles represent the twin pairs, while the black circles refer to pairs of unrelated individuals. Diagonal is equality line. Error bars show one standard deviation.
(PDF)

S5 Fig Number of shared in-frame alpha TCR CDR3 clonotypes reported between all 15 pairs of 6 donors consisting of 3 twin pairs (ordinate) compared to the model prediction (abscissa). To be able to compare datasets of different sizes, the sharing number was normalized by the product of the two cloneset sizes. The three red circles represent the twin pairs, while the black circles refer to pairs of unrelated individuals. Diagonal is equality line.
(PDF)

S6 Fig Mean number of insertions in shared sequences in alpha out-of-frame repertoires. (PDF)

S7 Fig Mean number of insertions in shared sequences in alpha out-of-frame repertoires of CD45RO+ (memory) cells. (PDF)

S8 Fig Reproducibility of our results using previously published data. Distribution of *P*_gen_—the probability that a sequence is generated by the VJ recombination process—for shared out-of-frame TCR alpha clonotypes between individual *A*_1_ from [8] and the other five individuals. While the distribution of shared sequences between unrelated individuals (red curves) is well explained by coincidental convergent recombination as predicted by our stochastic model (blue curve), sequences shared between two twins (green curve) have an excess of low probability sequences: 68 sequences with log_10_ *P*_gen_ < −10. For comparison the distribution of *P*_gen_ in regular (not necessarily shared) sequences is shown in black.
(PDF)

S9 Fig Normalized sharing of out-of-frame zero insertion clonotypes. Number of shared out-frame alpha zero insertion TCR CDR3 clonotypes reported between all 15 pairs of 6 donors consisting of 3 twin pairs (ordinate) compared to the model prediction (abscissa). The three red circles represent the twin pairs, while the black circles refer to pairs of unrelated individuals. Diagonal is equality line. Error bars show one standard deviation.
(PDF)

S10 Fig Dependence of mean insertion number on rank holds for different bin sizes. Mean numbers of insertions were obtained by analysing subsequent groups of 1000 (A) and 4000 (B) sequences of decreasing abundances, as in Fig 3A from the main text. (C,D,E) are results for ageing datasets reproduced for the top 1000, 2000 and 4000 clonotypes. Solid lines are independent fits to exponential decays (see main text Methods). Decay rate parameters for top 1000 and top 4000 clones are 0.0218 yr^−1^ and 0.0184 yr^−1^ respectively, within one standard error of the estimate for the top 2000 clones, 0.0272 ± 0.0091 yr^−1^.
(PDF)

S11 Fig The dependence between clone abundance and mean insertion number is robust across cord blood donors. Mean numbers of insertions were obtained by analysing groups of 3000 sequences of decreasing abundances as in Fig 3A, for 7 independent published cord blood samples from [24]. A similar decreasing trend is observed for all samples.
(PDF)

S12 Fig Rank-abundance dependencies. Here we show the dependence of the clone abundance on its abundance rank in samples from Fig 3A. Memory clones are typically larger than the naive and cord blood clones of same rank, possibly due to the history of clonal expansions.
(PDF)

S1 Table List of primers used. (XLSX)

S2 Table Number of reads, UMI and unique CDR3 nucleotide sequences in each sample. (XLSX)

S3 Table Ageing data used for Fig 4 and exponential decay fits. Percentage of the naive T-cells defined using flow cytometry, see [23] for details.
(XLSX)
