# Supplementary material for: Persisting fetal clonotypes influence the structure and overlap of adult human T cell receptor repertoires
Source: PLoS Comput Biol. 2017 Jul 6;13(7):e1005572. doi: 10.1371/journal.pcbi.1005572 (PMC5500008; doi:10.1371/journal.pcbi.1005572)
Supplement: S1 Text — (PDF) [file pcbi.1005572.s001.pdf]

I. SUPPLEMENTARY MATERIALS AND METHODS

A. Blood samples

Blood samples were collected from 3 pairs of monozygotic twin female donors, 23 (donors S1 and S2), 23 (donors P1 and P2) and 25 (donors Q1 and Q2) years old respectively. The individuals in each twin pair lived together for most of their lives, they were also tested for absence of dangerous infections before working with their blood (e.g. Hep C, HIV, syphilis). We also collected blood from two 19 and 57 year old male donors, along with a 51 year old female donor for memory and naive T-cells isolation, and a cord blood sample from a female newborn. All donors were healthy Caucasians, blood samples were collected with informed consent, and local ethical committee approval. The genetic identity of the twins was checked using polymorphic Alu insertion genotyping [1].

PBMCs were isolated from 12 ml of blood using Ficoll-Paque (Paneco, Russia) density gradient centrifugation. One third of the isolated PBMCs was used for total RNA isolation with the Trizol reagent (Invitrogen, USA) according to the manufacturer's protocol. Other cells were used for CD4, CD8 and CD45RO+ T-cells isolation.

B. CD4, CD8, 45RO+ T-cell isolation

CD4 and CD8 T-cells were isolated from PBMCs using the CD4+ and CD8+ positive selection kit (Invitrogen, USA) according to the manufacturer's protocol. CD8 T-cells were isolated from CD4 depleted samples to maximize the cell yield. 45RO+ cells were extracted using human CD45RO microbeads (Myltenyi, USA). Naive T-cells were isolated with the CD8+ T-cell naive isolation kit (Myltenyi, USA) according to the manufacturer's protocol without the final CD8 enrichment step.

Total RNA was immediately extracted from the isolated cells using the Trizol reagent (Invitrogen).

C. TCR α and TCR β cDNA library preparation

The library preparation protocol was adapted from [2] with modifications. The cDNA first strand was produced from the total RNA using the SmartScribe kit (Clontech, USA) and universal primers specific for the C-segment (see Fig. S1 A). Custom cap-switching oligonucleotides with unique molecular identifiers (UMI) and sample barcodes were used to introduce the universal primer binding site to the 3' end of the cDNA molecules (see Fig. S1 B). Each tube contained 500 ng of total RNA (corresponding to approximately 500000 PBMCs), 1x SmartScribe buffer, dNTP (1 mM each), 10pcmol of BCuniR4vvshort and TRACR2 primers (see Table S1 for sequences) and 1 μl of SmartScribe reverse transcriptase. 5mkg of the total RNA was used for the cDNA synthesis for each sample (10 tubes per sample, corresponding to approximately 5000000 PBMCs). The cDNA synthesis product was treated (45 min, 37°C) with 1 μl of 5u/μl UDG (NEB, USA) to digest the cap-switching oligonucleotide and purified with the Quigen PCR purification kit. After the cDNA synthesis two steps of PCR amplification were used to amplify the cDNA and also introduce Illumina TruSeq adapters as well as the second sample barcode. After both steps the PCR product was purified using the Quigen PCR purification kit according to the manufacturer's protocol. The first PCR step (see Fig. S1 C) consists of 16 cycles of: 94 °C for 20 sec, 60°C for 15 sec, 72°C for 60 sec. Each tube contained (total reaction volume 15 μl) 1x Q5 polymerase buffer (NEB), 5 pmol of Sm1msq and RPbcj1, RPbcj2, RPacj primers, dNTP(0.125 mM each) and 0.15 μl of Q5 polymerase. Then 1 μl of the purified PCR product was used for the second amplification step (see Fig. S1 D) consisting of 12 cycles of: 94°C 20 sec, 60°C 15 sec, 72°C 40 sec. Each tube contained (total reaction volume 25 μl): 1x Q5 polymerase buffer, 5 pmol of Smoutmsq and Il-bcj-ind or Il-acj-ind primers (with sample specific indices, for beta and alpha libraries respectively, one primer per sample), dNTP(0.125 mM each) and 0.25 μl of Q5 polymerase. Size selection for 500-800bp fragments of the purified PCR product was performed using electrophoresis in 1% agarose gel.

D. Next Generation Sequencing

cDNA libraries were sequenced on the Illumina HiSeq platform (2x100nt). Custom sequencing primer sequences are listed in Table S1. The total numbers of sequencing reads are shown in Table S2.

E. Raw data preprocessing

All raw datasets used in this study are available online. For details about the donors see SI Materials and Methods Section A.

Twin TCR alpha chain sequences (3 identical twin pairs):
`https://files.pub.cdr3.net/pogorely/HtSyudY2lkJ78TgzUKEshYUj4/alpha.tar`

Twin TCR beta chain sequences (3 identical twin pairs):
`https://files.pub.cdr3.net/pogorely/HtSyudY2lkJ78TgzUKEshYUj4/beta.tar`

Memory and naive cells TCR beta sequences for three donors aged 19, 51 and 57, and an unsorted cord blood sample:
`https://files.pub.cdr3.net/pogorely/HtSyudY2lkJ78TgzUKEshYUj4/mem_naive_cord.tar`

Sample sheet containing barcode sequences and filenames of the samples:

https://docs.google.com/spreadsheets/d/1YTBXYP8ITpaVkUx46s_DtfBlZfvIu6UdGjcde-csMy4

Sequencing data from individuals of different ages used in Fig. 4 is publicly available in the SRA:

http://www.ncbi.nlm.nih.gov/sra/PRJNA316572

Raw sequencing data files were preprocessed with MiGEC [3], sequencing reads were clustered by unique molecular identifiers (UMI). UMIs with less than two reads were discarded to reduce the number of erroneous sequences. Then sequences were processed with MiXCR [4] to determine the CDR3 position and nucleotide sequence. For the numbers of UMIs after filtering see Table S2.

F. Learning recombination statistics

We built a generative model that describes the probability of generation of recombined sequences, following the theoretical framework described in [5–7]. The generation probability for each sequence is calculated as the sum over all recombination scenarios r that can produce that sequence, $P_{\mathrm{gen}}(\mathrm{sequence}) = \sum_r P_{\mathrm{rearr}}(r)$. For TCR alpha chains the model assumes the following factorized form for a recombination scenario defined by the choice of genes (V and J), $P(V, J)$, deletions (delV and delJ), $P(\mathrm{del}V|V)$ and $P(\mathrm{del}J|J)$ and insertions (ins), $P(\mathrm{ins})$:

$$P^{\alpha}_{\mathrm{rearr}}(r) = P(V, J)P(\mathrm{del}V|V)P(\mathrm{del}J|J)P(\mathrm{ins}). \quad (1)$$

The parameters of the models, the different probabilities in the factorized formula, were inferred by maximizing the likelihood of the observed out-of-frame sequences given the model, using Expectation-Maximization [5]. For alpha chains, the model was reformulated as a Hidden Markov Model, and the parameters were learned efficiently using a Baum-Welch algorithm, as described in [6].

For beta chains, the model describes probabilities for V, D and J choices, with possible deletions and insertions at each of the two junctions:

$$\begin{aligned} P^{\beta}_{\mathrm{rearr}}(r) &= P(V, D, J)P(\mathrm{del}V|V)P(\mathrm{ins}VD) \quad (2)\\ &\quad \times P(\mathrm{del}Dl, \mathrm{del}Dr|D)P(\mathrm{ins}DJ)P(\mathrm{del}J|J) \end{aligned}$$

The parameters for the beta chain model were inferred directly using the Expectation-Maximization algorithm, by enumerating all possible recombination scenarios that can produce each sequence, using the procedure described in [5, 7].

This procedure allows us to learn the features of the recombination statistics with great accuracy, in particular the distribution of number of insertions at the junctions, even though the recombination events themselves cannot be unambiguously be determined for each sequence because of convergent recombination.

G. Distribution of insertions for each beta chains abundance class

We applied the procedure described in the previous section separately for each abundance class of the beta-chain sequences. However, given the small size of the datasets (2000 or 3000 sequences), we did not learn the full model for each class. Instead, we used a previously inferred universal beta-chain recombination model [5] for the V,D,J gene usages and their deletion profiles, and we learned the insertion distributions ($P(\mathrm{ins}VD)$ and $P(\mathrm{ins}DJ)$) for each class separately, while keeping the other parameters constant. The distribution of insertions thus inferred are used to plot the results of Figs. 3 and 4 of the main text.

It should be noted that the effect size depends on the bin size. We replicated our analysis with different bin sizes, to show that the effect is still present (see Fig. S10). Larger bins lead to lower effect sizes, but also to lower errors, so the significance of the difference in number of insertions between abundant and non-abundant clones is robust to the choice of the bin size.

To show that our results are not specific to certain donors, we reproduced our results shown on Fig. 3A for 7 additional published cord blood repertoires from [8], see Fig. S11. All mean insertion distributions in all samples follow the same trend as the one presented on Fig. 3.

We also show how abundance varies with ranks inside each sample presented on Fig. 3A on Fig. S12. Memory clones are typically more abundant than naive clones in same the individual, as was previously described [9]. The high frequencies of the few most abundant naive clones could be explained by contamination with memory compartment on the magnetic column. More accurate naive-memory separation method could potentially enhance the effect seen in Fig. 3A.

In Fig. 4 we show the decay of zero-insertion clonotypes from the 2000 most abundant clones in unsorted TCR repertoires from a published dataset of donors of various ages [8]. We hypothesise that the observed decay is due not only to the decay of naive pool, but also to the decay of fetal clones within the naive pool. However, a possible dramatic difference in the naive-memory partition of these abundant clones could confound this effect. To exclude this possibility, we estimated the naive-memory composition of 2000 most abundant clones from the unpartitioned, naive, and memory datasets of the three donors presented on Fig. 3A, who are of different ages. We attribute a clonotype from the unpartitoned dataset to the memory pool if the rank of this clone in the memory dataset was higher than in the naive one. We show that the ratio of naive to memory clonotypes in the 2000 most abundant clones is similar among all 3 donors, and is not decaying significantly with age: 1159 memory to 767 naive for the 19 year old donor (74 clones have undetermined phenotype), 1313 memory to 686 naive for the 57 year old donor (1 clone has undetermined phenotype) , and 1128 memory to 858 naive (14 clones have

undetermined phenotype) for the 51 year old donor.

H. Inference of selection factors

In-frame sequences statistically differ from out-of-frame sequences (besides their frameshift), because in-frame sequences are functional and have passed thymic selection. For each sequence we defined a selection factor Q as the ratio of the probability of observing the sequence in the in-frame set, to the probability of recombining the sequence according to out-of-frame statistics (as inferred above). The overal selection factor Q is assumed to be the product of several independent factors q acting on the CDR3 length L and on the identity of amino acid a_i at each position i of the CDR3 [10]:

$$Q \propto q_L \prod_{i=1}^{L} q_{i;L}(a_i) \tag{3}$$

The parameters were inferred by maximizing the likelihood with gradient ascent, as described in [10].

I. Data analysis

Analysis of the shared clonotypes was performed using the R statistical programming language [11] and the tcR package [12].

J. Out-of-frame sharing prediction

To predict sharing for each individual, we generated sequences using our recombination model P_{gen} (alpha or beta), with individually inferred model parameters. Normalized sharing of the TCR sequences between two clonesets is defined as the number of the same unique TCR nucleotide sequences observed in both of them, divided by the product of the total numbers of unique TCR nucleotide sequences in the two datasets.

We calculated sharing of either whole chains, or of their CDR3, defined as the sub-sequence going from the conserved cystein at the end of the V region, to the conserved phenylalanine in the J region.

The alpha chain results for whole-chain sharing are plotted in the main text in Fig. 1, and the data shows good agreement with the model. The results for CDR3 sharing are shown in Fig. S2. The model systematically underestimates the normalized sharing by a common multiplicative factor of 1.7 for non-twins, with a Pearson correlation coefficient of 0.8 between the data and the model prediction. Absolute numbers of shared CDR3 sequences for alpha chains varied from 400 to 1200.

For beta chain sequences, the prediction of out-of-frame sharing is more difficult because of the low numbers of out-of-frame sequences in the RNA data, which, combined to a lower mean P_{gen}, results in a much lower number of shared out-of-frame sequences. We also identified and removed from the dataset 26 out-of-frame sequences shared between more than two individuals. These sequences are likely to arise due to reproducible aligner errors or technology artifacts – some of them contained intronic sequences, etc. Absolute numbers of shared beta CDR3 sequences varied from 0 to 82. Nevertheless, the number of shared beta out-of-frame CDR3 sequences for twins exceeded the model prediction (see Fig. S3), confirming our hypothesis of biological contamination during pregnancy.

K. In-frame sharing prediction

To accurately predict the normalized sharing number for in-frame nucleotide clonotypes, we generated sequences from P_{gen} as we did for out-of-frame sequences, but weighted them by their selection factor Q to account for thymic selection. The predicted normalized sharing number was then calculated as:

$$\frac{1}{|S_1| \cdot |S_2|} \sum_{s \in S_1 \cap S_2} Q^{(1)}(s) Q^{(2)}(s), \tag{4}$$

where S_1, and S_2 are two synthetic sequence samples drawn from two models $P_{\text{gen}}^{(1)}$,$P_{\text{gen}}^{(2)}$ individually learned from the out-of-frame sequences of two individuals, and $Q^{(1)}(s)$, $Q^{(2)}(s)$ are selection factors learned individually from these two individuals' in-frame sequences. $|S_1|$ and $|S_2|$ denote the size of the two samples. The sum runs over sequences s found in both samples.

For both the beta and the alpha chains, the prediction agrees very well with the data (Fig. S4 and Fig. S5). For the beta chain, twins share more CDR3 sequences than non-twin pairs, while no such effect was observed for the alpha chain sequences. This fact could be explained by the much higher number of clonotypes shared due to convergent recombination in the alpha in-frame dataset than in the beta in-frame and alpha and beta out-of-frame datasets. Excess of shared CDR3 nucleotide sequences due to biological contamination in twins is lower than the amount of convergent recombination noise in the alpha in-frame shared CDR3 nucleotide sequences. Absolute numbers of shared in-frame CDR3 sequences for alpha chains varied from 30000-50000 sequences depending of the pair, and 5000-9000 for beta chains.

L. Mixed model inference

We hypothesized that the larger amount of zero insertion clonotypes is responsible for the increase in sharing between the most abundant clonotypes of the out-of-frame repertoires of unrelated individuals. To test this hypothesis, we constructed a mixture model for each

abundance class, each class containing 2000 clonotypes ranked by decreasing abundance.

We assume that abundance class C contains a fraction $F(C)$ of clonotypes generated with zero insertions, and $1 - F(C)$ of regular clonotypes. Obtaining $F(C)$ is not straightforward because regular clonotypes can also zero insertions. In addition, the number of insertions cannot be determined with certainty – for example, a deletion followed by an insertion matching the germline sequence can be wrongly interpreted as a case of no insertions.

To circumvent this problem, we determine for each abundance class a simpler quantity to estimate, namely the fraction $F_0(C)$ of clonotypes that are consistent with zero insertions, *i.e.* that can be entirely matched to the germline genes. Because of the reasons outlined above, $F_0(C)$ is *not* equal to $F(C)$. However, $F_0(C)$ is a linear function of $F(C)$, $F_0(C) = A + BF(C)$. Therefore, if we can generate synthetic sequences such that their $F_0(C)$ agrees with data, then we are guaranteed that their $F(C)$ will coincide with the data as well, even if we do not know the explicit mixing parameters $F(C)$.

To obtain this mixture, we generated many sequences from our recombination model P_{gen}. To determine which generated sequences were consistent with zero insertions, we aligned them to all possible V and J genomic templates. We then separated out the sequences consistent with zero insertions from the others, and created, for each abundance class C, and artificial dataset with a fraction $F_0(C)$ of such sequences, and $1 - F_0(C)$ of the other sequences (not consistent with zero insertions), where $F_0(C)$ is given by the data.

We then calculated normalized sharing in the synthetic data by including an increasing number of abundance classes, starting with the most abundant ones, and compared to data in Fig. 5.

II. SUPPLEMENTARY RESULTS

A. Distinctive properties of shared clonotypes between twins

Shared clonotypes in unrelated individuals appear in the process of convergent recombination. Sequences with a higher P_{gen} are thus more likely to be shared, and we can calculate accurately the distribution of P_{gen} among shared sequences (see Fig. 2). We observe that sequences shared between twins violate this prediction, consistent with our hypothesis that some of these sequences are due to biological contamination. To confirm this, we used a sequence feature that is negatively correlated with P_{gen} [5]: the number of insertions in the CDR3 region. The number of insertions in CDR3 sequences shared between unrelated individuals was indeed lower (Fig. S6) than the mean number of insertions in non-shared sequences. However, the mean number of insertions in sequences shared between twins (black boxes) is higher than in unrelated individuals, $p = 1.83 \cdot 10^{-8}$, two-sided t-test. The same and even stronger effect is observed for memory (CD45RO+) cells, $p < 10^{-16}$, two-sided t-test (Fig. S7).

Our theory also predicts that twins should have an excess of zero-insertion shared clonotypes, relative to non-twins. To check for this, we compared the normalized sharing number of zero-insertion out-of-frame clonotypes in the data and according to the model (see Fig. S9). Although we observe higher sharing numbers in twins, this effect is made non-significant by high levels of noise. Since zero-insertion clonotypes have low diversity, these normalized sharing numbers are much higher than their generic counterpart of Fig. 1. In other words, convergent recombination is much more likely, masking the effects of fetal contamination.

Finally, the mean clone size of low-probability ($P_{\text{gen}} < 10^{-10}$), twin-shared sequences from Fig. 2, 8.8 ± 0.7, is significantly larger than that of generic low-probability ($P_{\text{gen}} < 10^{-10}$) clones from that individual, 1.83 ± 0.013, providing another evidence of their fetal origin.

B. The phenotype of beta chain out-of-frame shared clonotypes

Two individuals displayed the most prominent excess of shared beta out-of-frame sequences. Since the model prediction for the number of shared sequences is close to zero we suppose that most of these shared sequences did not arise due to convergent recombination. These out-of-frame clones bear a second functional allele (otherwise they would have been filtered by selection in a thymus), and they also should have either the CD4 or the CD8 phenotype. To attribute these clonotypes a phenotype we separately sequenced CD4, CD8 and CD45RO positive subsets for the two donors and searched for the 84 out-of-frame CDR3s shared between the unpartitioned out-of-frame repertoires. 44 CDR3s were found in the CD8 subsets of both individuals, and only 5 sequences were found in the CD4 subsets of both individuals. 25 out of the 44 CD8 and 3 out of the 5 sequences were also found in the 45RO+ compartment. Only 3 sequences were mapped discordantly (e.g. CD4 in one twin and CD8 in the second twin), and 2 sequences were absent from the CD4, CD8 and CD45RO compartments of both individuals. For the other 32 sequences the CD4/CD8 status could be determined only for one individual (most probably due to the sequencing depth limitations). In case of convergent recombination it is unlikely that shared nonproductive sequences would have the same phenotype in different donors. The phenotypic study thus confirms the biological contamination hypothesis.

C. Our results are reproducible using previously published data

We tested the robustness of our results on previously published twin data from [13]. We observed the same

excess of low-probability shared sequences in twins compared to unrelated individuals as in Fig. 2 (see Fig. S8). These data also allowed us to control for possible experimental contamination. One of the twin pairs that participated in the present study was sequenced three years ago, using a different technology described in [13], excluding the possibility of any contamination between the old and new samples. Out of 84 beta out-of-frame clonotypes shared between two new twin samples, 59 were also shared between the new sample of one twin, and the old sample of the second twin. Therefore the out-of-frame sequences shared between the twins are reproducible and could not be result of experimental contamination with PCR-products or RNA.

D. Invariant T-cell alpha clonotypes in the data

It was previously shown that mucosal-associated invariant T-cells (MAIT) and natural killer T-cells (NKT) have an invariant alpha chain with very low diversity [14]. Specific V-J combinations are chosen (TRAV10/TRAJ18 for NKTs and TRAV1-2/TRAJ33 for MAIT) and no nucleotides are inserted in the recombination process of these clonotypes. To see whether these clonotypes could potentially confound our analysis, we searched for published NKT and MAIT sequences in our datasets. 25 out of the 27 known MAIT sequences were found in the datasets at least once (21 out of them in the all six individuals), and 8 out of the 13 known NKT sequences (2 of them in the all six individuals). MAIT and NKT sequences are present in our data, but only a few shared sequences could be explained by them, so we do not exclude MAIT and NKT alpha sequences from the analysis. The majority of shared zero insertion sequences could thus not be attributed to known MAIT or NKT subsets.

[1] Mamedov IZ, et al. (2010) A new set of markers for human identification based on 32 polymorphic Alu insertions. *European journal of human genetics : EJHG* 18:808–14.

[2] Mamedov IZ, et al. (2013) Preparing unbiased T-cell receptor and antibody cDNA libraries for the deep next generation sequencing profiling. *Frontiers in immunology* 4:456.

[3] Shugay M, et al. (2014) Towards error-free profiling of immune repertoires. *Nature methods* 11:653–5.

[4] Bolotin DA, et al. (2015) MiXCR : software for comprehensive adaptive immunity profiling. *Nature methods* 12:380–381.

[5] Murugan A, Mora T, Walczak AM, Callan CG (2012) Statistical inference of the generation probability of T-cell receptors from sequence repertoires. *Proc. Natl. Acad. Sci.* 109:16161–16166.

[6] Elhanati Y, Marcou Q, Mora T, Walczak AM (2016) repgenhmm: a dynamic programming tool to infer the rules of immune receptor generation from sequence data. *Bioinformatics* 32:1943–1951.

[7] Marcou Q, Mora T, Walczak AM (2017) IGoR: a tool for probabilistic high-throughput immune repertoire analysis. *In preparation.*

[8] Britanova OV, et al. (2016) Dynamics of Individual T Cell Repertoires: From Cord Blood to Centenarians. *J. Immunol.* 196:5005–5013.

[9] Venturi V, et al. (2011) A mechanism for TCR sharing between T cell subsets and individuals revealed by pyrosequencing. *J. Immunol.* 186:4285–4294.

[10] Elhanati Y, Murugan A, Callan CG, Mora T, Walczak AM (2014) Quantifying selection in immune receptor repertoires. *Proc. Natl. Acad. Sci.* 111:9875–9880.

[11] R Core Team (2014) *R: A Language and Environment for Statistical Computing* (R Foundation for Statistical Computing, Vienna, Austria).

[12] Nazarov VI, et al. (2015) tcR: an R package for T cell receptor repertoire advanced data analysis. *BMC bioinformatics* 16:175.

[13] Zvyagin IV, et al. (2014) Distinctive properties of identical twins' TCR repertoires revealed by high-throughput sequencing. *Proc. Natl. Acad. Sci. U. S. A.* 111:5980–5.

[14] Greenaway HY, et al. (2013) NKT and MAIT invariant TCRα sequences can be produced efficiently by VJ gene recombination. *Immunobiology* 218:213–24.

[15] Britanova OV, et al. (2014) Age-Related Decrease in TCR Repertoire Diversity Measured with Deep and Normalized Sequence Profiling. *The Journal of Immunology* 192:2689–2698.

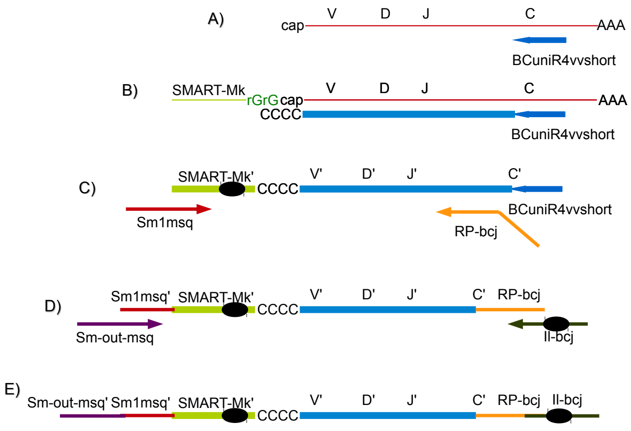

FIG. S1: **Library preparation protocol.** A) cDNA first strand synthesis for alpha and beta chains starts from specific primers in the C-segment conserved region. B) The template switching effect was used to introduce a universal primer binding site to the 3'cDNA end. The SMART-Mk sequence contains a sample barcode (black ellipse) for contamination control. C) and D) In two subsequent PCR steps we introduce the TruSeq adapter sequences along with Illumina sample barcodes (black ellipse). E) The resulting cDNA molecule is double barcoded, contains a Unique Molecular Identifier (UMI) and is suitable for direct sequencing on the Illumina HiSeq platform with the custom primers.

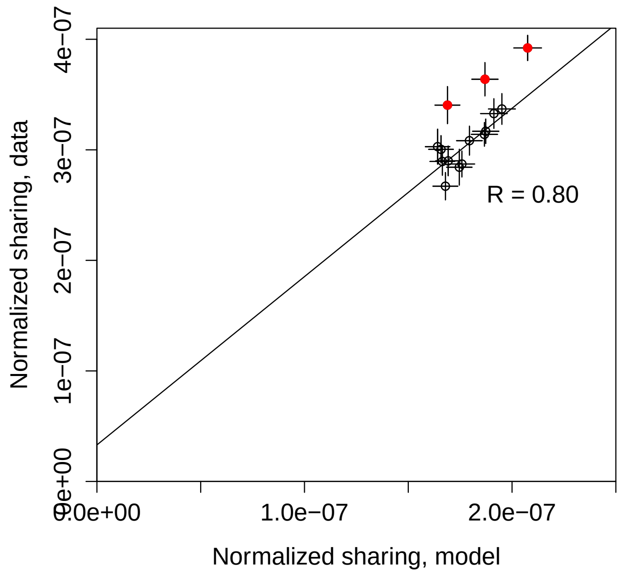

FIG. S2: Number of shared out-of-frame alpha TCR CDR3 clonotypes reported between all 15 pairs of 6 donors consisting of 3 twin pairs (ordinate) compared to the model prediction (abscissa). To be able to compare datasets of different sizes, the sharing number was normalized by the product of the two cloneset sizes. The outlying three red circles represent the twin pairs, while the black circles refer to pairs of unrelated individuals. Error bars show one standard deviation. The diagonal line is a linear fit for unrelated individuals, of slope 1.7.

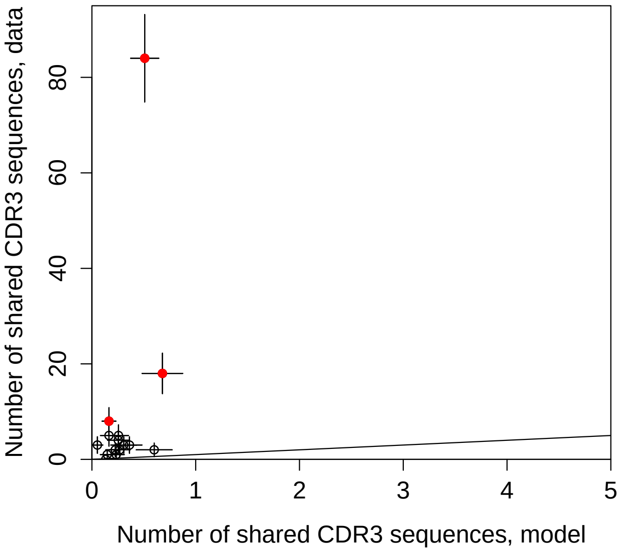

FIG. S3: Number of shared out-frame beta TCR CDR3 clonotypes reported between all 15 pairs of 6 donors consisting of 3 twin pairs (ordinate) compared to the model prediction (abscissa). The three outlying red circles represent the twin pairs, while the black circles refer to pairs of unrelated individuals. Error bars show one standard deviation.

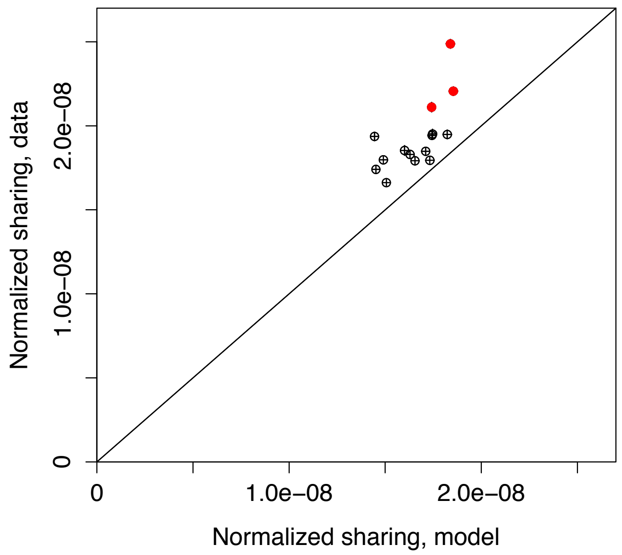

FIG. S4: Number of shared in-frame beta TCR CDR3 clonotypes reported between all 15 pairs of 6 donors consisting of 3 twin pairs (ordinate) compared to the model prediction (abscissa). To be able to compare datasets of different sizes, the sharing number was normalized by the product of the two cloneset sizes. The three outlying red circles represent the twin pairs, while the black circles refer to pairs of unrelated individuals. Diagonal is equality line. Error bars show one standard deviation.

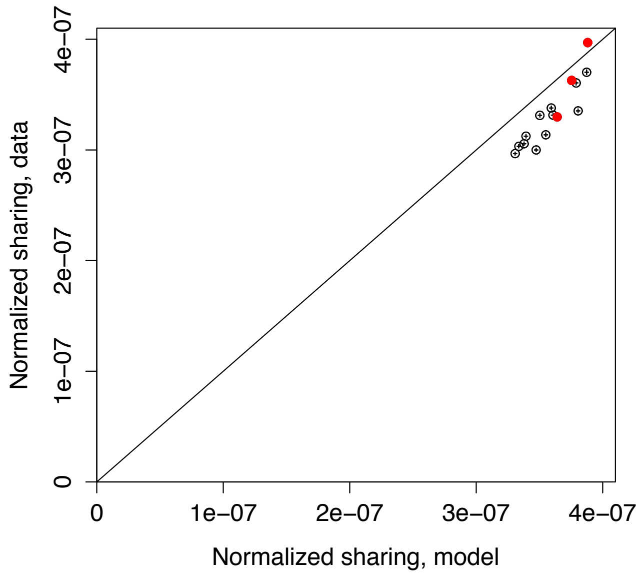

FIG. S5: Number of shared in-frame alpha TCR CDR3 clonotypes reported between all 15 pairs of 6 donors consisting of 3 twin pairs (ordinate) compared to the model prediction (abscissa). To be able to compare datasets of different sizes, the sharing number was normalized by the product of the two cloneset sizes. The three red circles represent the twin pairs, while the black circles refer to pairs of unrelated individuals. Diagonal is equality line.

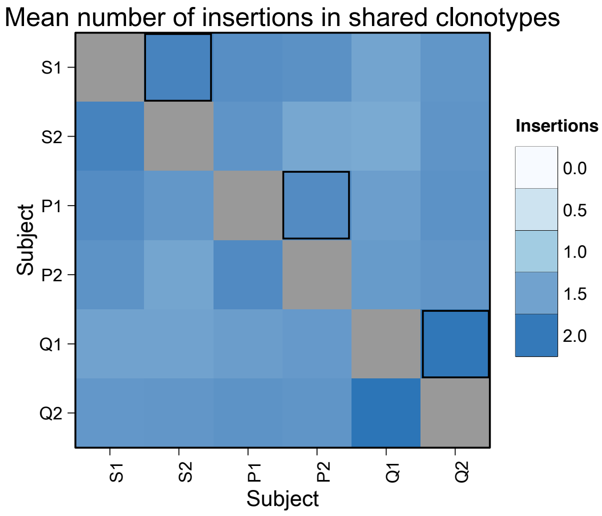

FIG. S6: Mean number of insertions in shared sequences in alpha out-of-frame repertoires.

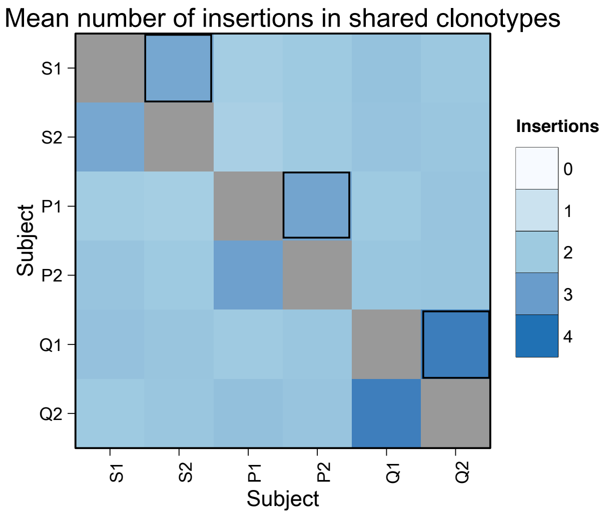

FIG. S7: Mean number of insertions in shared sequences in alpha out-of-frame repertoires of CD45RO+ (memory) cells.

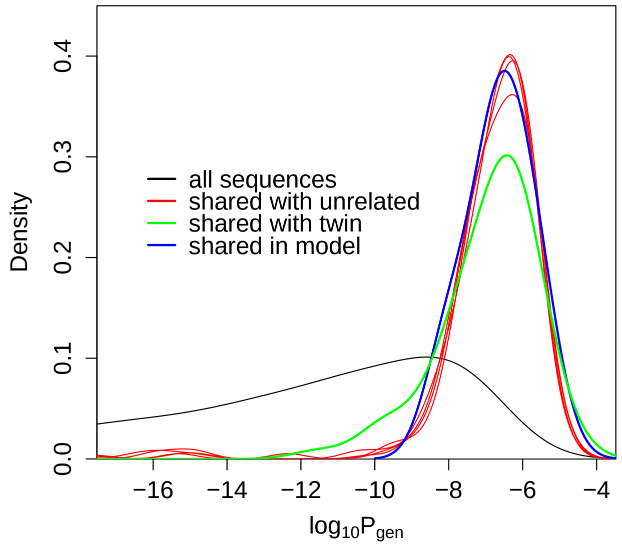

FIG. S8: **Reproducibility of our results using previously published data.** Distribution of P_{gen} – the probability that a sequence is generated by the VJ recombination process – for shared out-of-frame TCR alpha clonotypes between individual A_1 from [13] and the other five individuals. While the distribution of shared sequences between unrelated individuals (red curves) is well explained by coincidental convergent recombination as predicted by our stochastic model (blue curve), sequences shared between two twins (green curve) have an excess of low probability sequences: 68 sequences with $\log_{10} P_{\mathrm{gen}} < -10$. For comparison the distribution of P_{gen} in regular (not necessarily shared) sequences is shown in black.

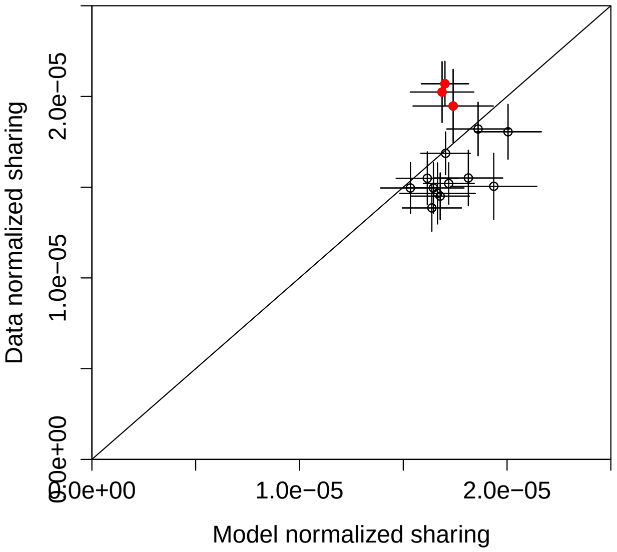

FIG. S9: **Normalized sharing of out-of-frame zero insertion clonotypes.** Number of shared out-frame alpha zero insertion TCR CDR3 clonotypes reported between all 15 pairs of 6 donors consisting of 3 twin pairs (ordinate) compared to the model prediction (abscissa). The three red circles represent the twin pairs, while the black circles refer to pairs of unrelated individuals. Diagonal is equality line. Error bars show one standard deviation.

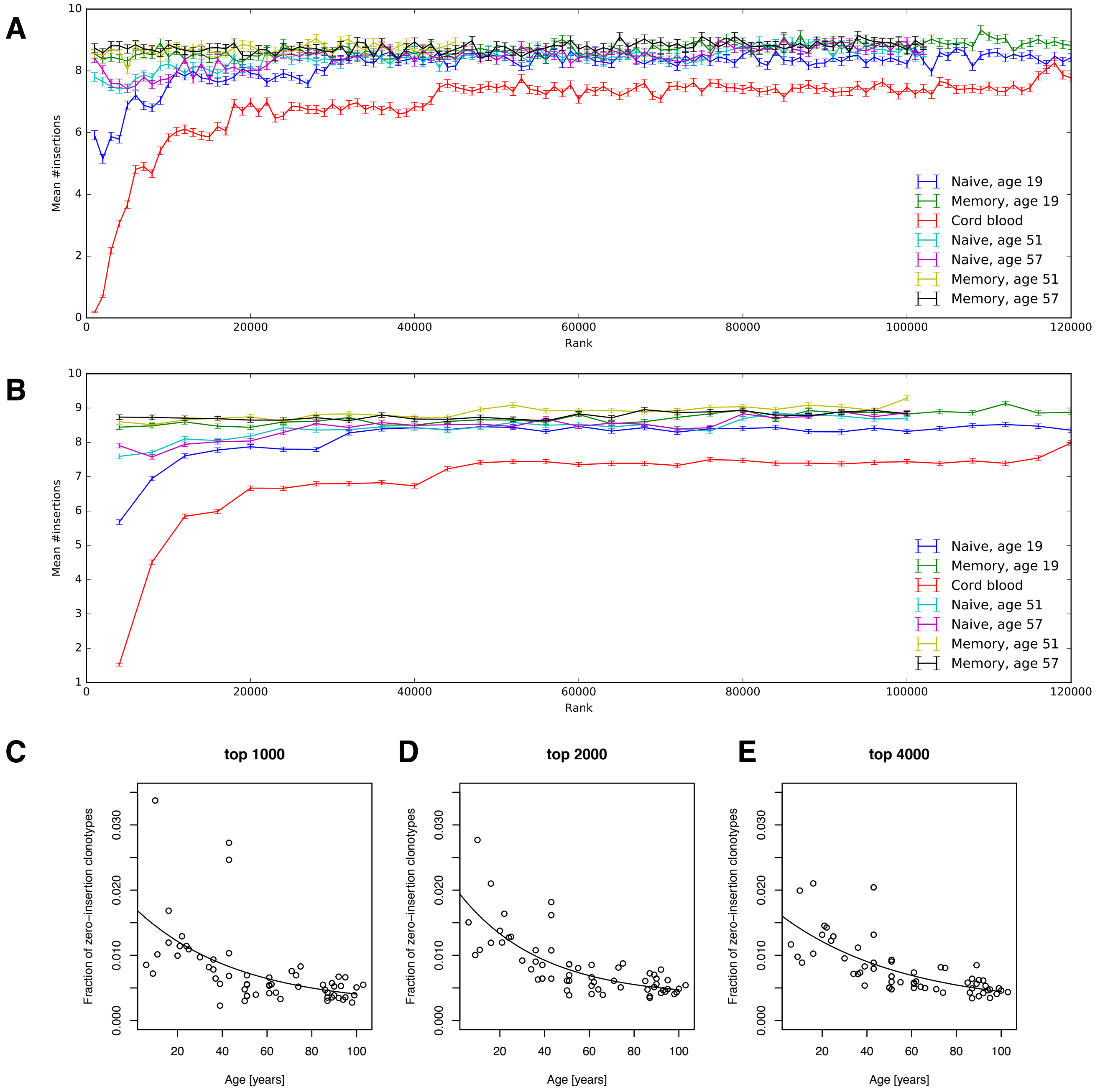

FIG. S10: **Dependence of mean insertion number on rank holds for different bin sizes.** Mean numbers of insertions were obtained by analysing subsequent groups of 1000 (A) and 4000 (B) sequences of decreasing abundances, as in Fig. 3A from the main text. (C,D,E) are results for ageing datasets reproduced for the top 1000, 2000 and 4000 clonotypes. Solid lines are independently fits to exponential decays (see main text Methods). Decay rate parameters for top 1000 and top 4000 clones are 0.0218 yr^{-1} and 0.0184 yr^{-1} respectively, within one standard error of the estimate for the top 2000 clones, 0.0272±0.0091 yr^{-1}.

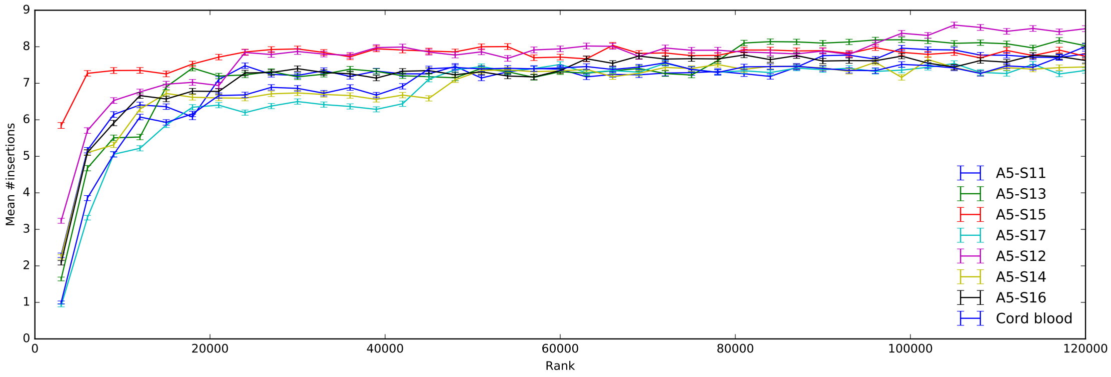

FIG. S11: **The dependence between clone abundance and mean insertion number is robust across cord blood donors.** Mean numbers of insertions were obtained by analysing groups of 3000 sequences of decreasing abundances as in Fig. 3A, for 7 independent published cord blood samples from [8]. A similar decreasing trend is observed for all samples.

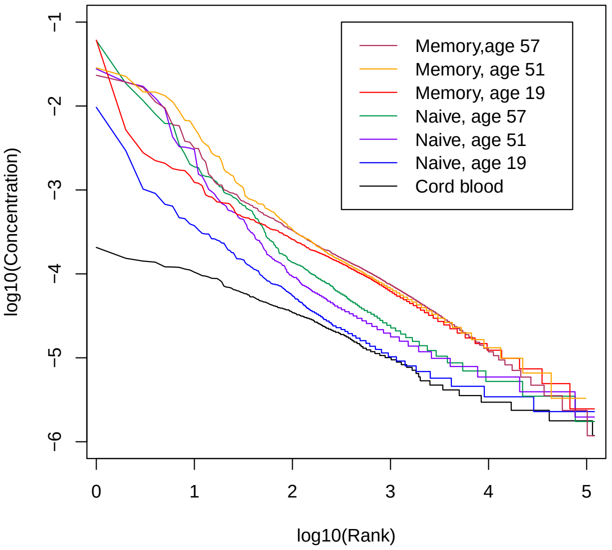

FIG. S12: **Rank-abundance dependencies.** Here we show the dependence of the clone abundance on its abundance rank in samples from Fig. 3A. Memory clones are typically larger than the naive and cord blood clones of same rank, possibly due to the history of clonal expansions.

SMART-Mk cap-switching oligonucleotides	
MK-108	CAGUGGUAUCAACGCAGAGUACNNNNNNUAATGCUNNNNNNUCTT(rG)(rG)(rG)(rG)
MK-248	CAGUGGUAUCAACGCAGAGUACNNNNUNNTGGCANNUNNNNNNUCTT(rG)(rG)(rG)(rG)
MK-253	CAGUGGUAUCAACGCAGAGUACNNNNUNNTTATGNNUNNNNNNUCTT(rG)(rG)(rG)(rG)
MK-103	CAGUGGUAUCAACGCAGAGUACNNNNNNUAACGGUNNNNNNUCTT(rG)(rG)(rG)(rG)
MK-257	CAGUGGUAUCAACGCAGAGUACNNNNUNNTTGCGNNUNNNNNNUCTT(rG)(rG)(rG)(rG)
MK-143	CAGUGGUAUCAACGCAGAGUACNNNNNNUCAGATUNNNNNNUCTT(rG)(rG)(rG)(rG)
MK-135	CAGUGGUAUCAACGCAGAGUACNNNNNNUATGCAUNNNNNNUCTT(rG)(rG)(rG)(rG)
MK-227	CAGUGGUAUCAACGCAGAGUACNNNNUNNTAACCNNUNNNNNNUCTT(rG)(rG)(rG)(rG)
cDNA synthesis primers	
BC_uni_R4vvshort	TGGAGTCATTGA
TRAC_R2	ACACATCAGAATCCTTACTTTG
PCR I step primers	
Sm1msq	GAGATCTACACGAGTCAGCAGTGGTATCAACGCAG
RPbcj1	CGACTCAGATTGGTACACCTTGTTCAGGTCCTC
RPbcj2	CGACTCAGATTGGTACACGTTTTTCAGGTCCTC
RPacj	CGACTCAAGTGTGTGGGTCAGGGTTCTGGATAT
PCR II step primers	XXXXXX stands for the Truseq index
Sm-out-msq	AATGATACGGCGACCACCGAGATCTACACGAGTCA
Il-bcj-indX	CAAGCAGAAGACGGCATACGAGATXXXXXXCGACTCAGATTGGTAC
Il-acj-indX	CAAGCAGAAGACGGCATACGAGATXXXXXXCGACTCAAGTGTGTGG
Custom sequencing primers	
IL-AIRP	ATATCCAGAACCCTGACCCACACACTTGAGTCG
IL-IRP-b1	GAGGACCTGAAAAACGTGTACCAATCTGAGTCG
IL-IRP-b2	GAGGACCTGAACAAGGTGTACCAATCTGAGTCG
IL-RP1-msq	ACACGAGTCAGCAGTGGTATCAACGCAGAGTAC
IL-RP2-b1	CGACTCAGATTGGTACACGTTTTTCAGGTCCTC
IL-RP2-b2	CGACTCAGATTGGTACACCTTGTTCAGGTCCTC
IL-ARP2	CGACTCAAGTGTGTGGGTCAGGGTTCTGGATAT

TABLE S1: List of primers used

Alpha chain			
Sample_id	Number of reads	Number of UMI	Number of unique CDR3nuc
P1_CD4	6566952	430915	248457
P1_CD8	4620425	378044	162607
P1_unpart	9571058	574439	348419
P1_45RO	4099026	431529	173883
P2_CD4	4269624	941176	432476
P2_CD8	4040615	561437	204094
P2_unpart	8213565	873546	471850
P2_45RO	4608991	653326	228429
Q1_CD4	3894188	653649	277621
Q1_CD8	3201067	589757	147918
Q1_unpart	8360990	1091786	456024
Q1_45RO	3587344	687916	201218
Q2_CD4	3877893	828573	315922
Q2_CD8	3880048	825539	158954
Q2_unpart	9159719	1215155	473672
Q2_45RO	3890664	834828	224276
S1_CD4	4655514	734158	360161
S1_CD8	1009038	219433	105232
S1_unpart	3191701	621723	351923
S1_45RO	4977466	495057	189739
S2_CD4	11727155	761495	348109
S2_CD8	12436797	468345	190534
S2_unpart	11135704	610105	336177
S2_45RO	9064981	633362	228579
Beta chain			
Sample_id	Number of reads	Number of UMI	Number of unique CDR3nuc
P1_CD4	3757755	759270	235040
P1_CD8	3565384	517737	204963
P1_unpart	7429601	955106	444708
P1_45RO	4036708	695379	195023
P2_CD4	3042278	449048	475545
P2_CD8	3438238	477696	241048
P2_unpart	8144134	817306	624074
P2_45RO	4598733	578663	249001
Q1_CD4	3694288	673037	386005
Q1_CD8	4586088	758201	237511
Q1_unpart	6511237	1060251	581114
Q1_45RO	3171012	664732	216879
Q2_CD4	3066472	605062	351640
Q2_CD8	3389029	691438	174552
Q2_unpart	7256515	1241753	644594
Q2_45RO	3110044	667997	214628
S1_CD4	3510759	722883	423689
S1_CD8	3162597	489393	248236
S1_unpart	7019324	1181194	673755
S1_45RO	3363725	574876	218185
S2_CD4	4034384	717023	410283
S2_CD8	4267632	546529	258832
S2_unpart	7093628	875357	521882
S2_45RO	2848644	526765	209807
Memory_aged19	7486248	424156	149292
Nave_aged19	9166800	932396	697091
Memory_aged51	4376542	366646	104477
Nave_aged51	4115592	602950	348005
Memory_aged57	5743372	476395	245092
Nave_aged57	5227973	358245	422545
Cord_blood	8015355	1803557	1119000

TABLE S2: Number of reads, UMI and unique CDR3 nucleotide sequences in each sample.

Sample id	fraction of 0 ins in top 2000	Naive,%	Age, years
A2-i132	0.015056135255	73.7	6
A2-i131	0.010037196444	43	9
A2-i136	0.027691639038	40	10
A2-i129	0.0108412940125	57	11
A2-i134	0.021007545075	68	16
A2-i133	0.0119257041822	60.9	16
A4-i194	0.013765206508	55	20
A4-i195	0.0119673129492	59	21
A4-i191	0.01637900271	45	22
A4-i192	0.012716977224	56	24
A4-i189	0.012839842368	44	25
A6-I201ob	0.0091925381272	NA	30
A3-i110	0.0078554903232	36.4	34
A3-i101	0.0107838068688	55	36
A4-i101	0.0090257537105	27	36
A4-i102	0.00628983345724	27.6	37
A3-i107	0.00851643362094	43	39
A4-i107	0.0064344051544	26	39
A3-i106	0.016159136094	39.4	43
A3-i102	0.0107591339774	27.3	43
A4-i110	0.018164859228	40	43
A4-i106	0.00642081990976	31	43
A5-S23	0.0046042762969	21.3	50
A5-S24	0.0061143105585	29.9	50
A6-I160	0.008621670788	38.9	51
A5-S21	0.0086245934928	51.3	51
A6-I215ob	0.00819076572358	NA	51
A5-S22	0.00695571384444	48.5	51
A6-I150	0.0061129801278	NA	51
A5-S20	0.00387005779589	25	51
A5-S19	0.0080402564192	41.2	55
A4-i185	0.0085319088075	29.6	61
A4-i186	0.00532914538306	14.6	61
A4-i184	0.00405847825812	21	61
A4-i188	0.00663226556694	18	61
A4-i128	0.0058717051432	23	62
A4-i125	0.00476704046791	4.5	64
A4-i124	0.00394006128853	16.3	66
A2-i141	0.0060990185169	30	71
A2-i140	0.0081195988401	47	73
A2-i138	0.00507840452028	6.7	74
A2-i139	0.008749966888	28.2	75
A4-i122	0.00606575047668	33	85
A3-i145	0.004749303571	37	86
A4-i132	0.0034771649962	14.5	87
A4-i183	0.00723588404502	24.6	87
A3-i150	0.0037069726895	13.3	87
A6-I214ob	0.0046188525124	21	88
A5-S10	0.007023235658	NA	89
A4-i118	0.00512286685575	54	89
A4-i127	0.005589445878	12.7	90
A5-S9	0.00642820638494	26.5	90
A6-I211ob	0.00432554146357	8.4	91
A5-S8	0.00421932231855	4.5	92
A5-S7	0.0078096377085	4.7	92
A6-I210ob	0.00368734455504	7.4	92
A6-I208ob	0.0045677109953	8.7	93
A5-S4	0.0046450251048	30.8	93
A6-I207ob	0.0044350512973	27.6	94
A6-I206ob	0.0061812657375	6.2	95
A6-I205ob	0.00481739413682	7.5	95
A5-S3	0.0040549739527	12.4	98
A6-I204ob	0.00431740407138	10.3	99
A5-S2	0.00486991171424	15.5	100
A5-S1	0.00541415235339	NA	103

TABLE S3: Ageing data used for Fig. 4 and exponential decay fits. Percentage of the naive T-cells defined using flow cytometry, see [15] for details.
