# Supplementary figures and images for: Persisting fetal clonotypes influence the structure and overlap of adult human T cell receptor repertoires
Source: PLoS Comput Biol. 2017 Jul 6;13(7):e1005572. doi: 10.1371/journal.pcbi.1005572 (PMC5500008; doi:10.1371/journal.pcbi.1005572)

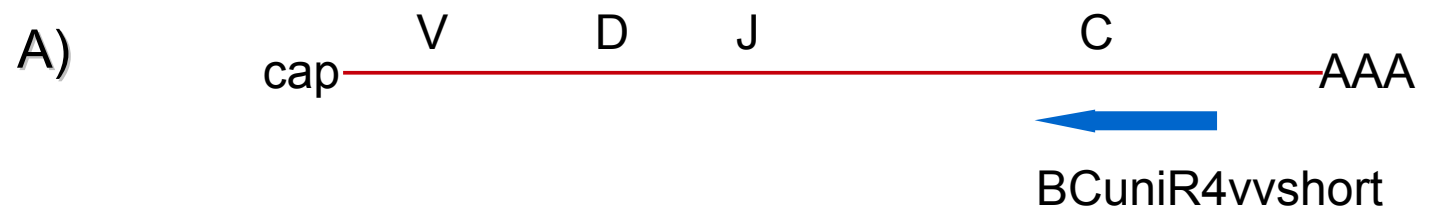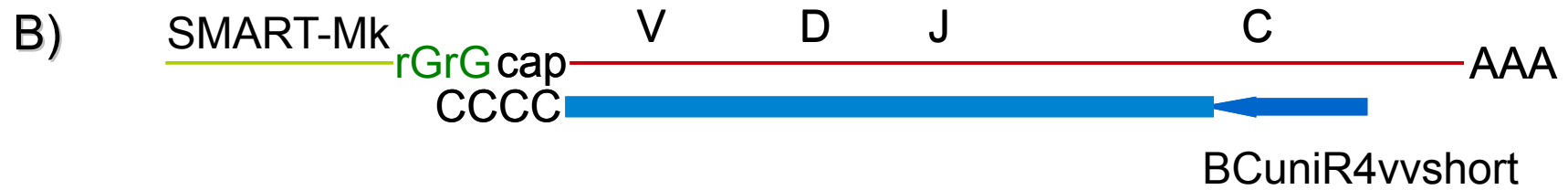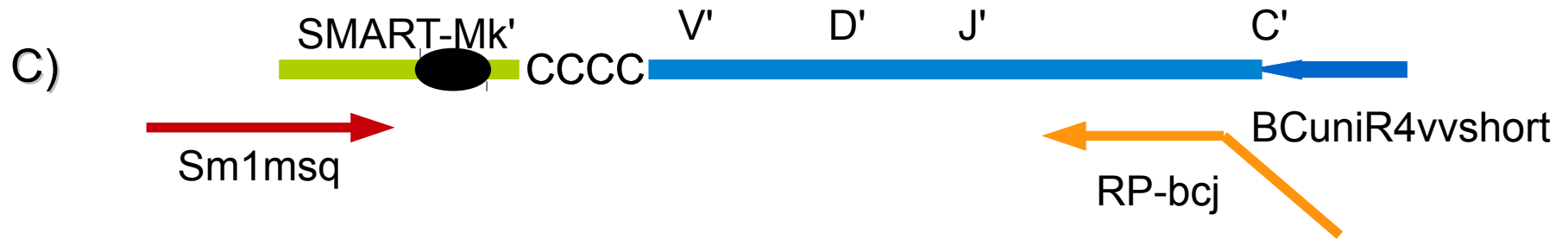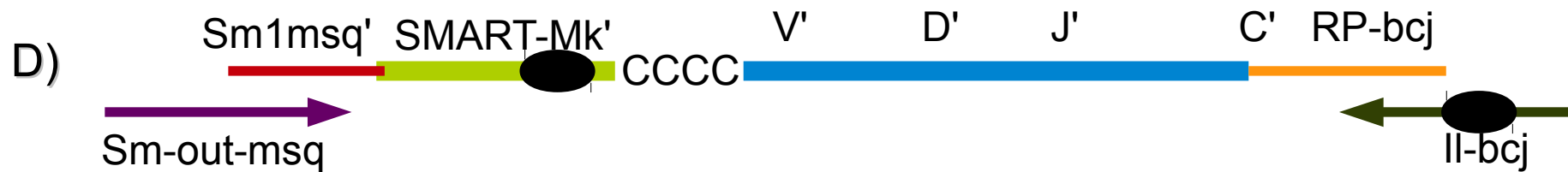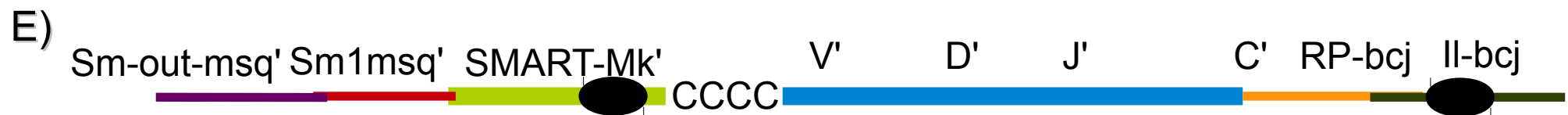

Supplement: S1 Fig — A) cDNA first strand synthesis for alpha and beta chains starts from specific primers in the C-segment conserved region. B) The template switching effect was used to introduce a universal primer binding site to the 3’cDNA end. The SMART-Mk sequence contains a sample barcode (black ellipse) for contamination control. C) and D) In two subsequent PCR steps we introduce the TruSeq adapter sequences along with Illumina sample barcodes (black ellipse). E) The resulting cDNA molecule is double barcoded, contains a Unique Molecular Identifier (UMI) and is suitable for direct sequencing on the Illumina HiSeq platform with the custom primers. (PDF) [file pcbi.1005572.s002.pdf]

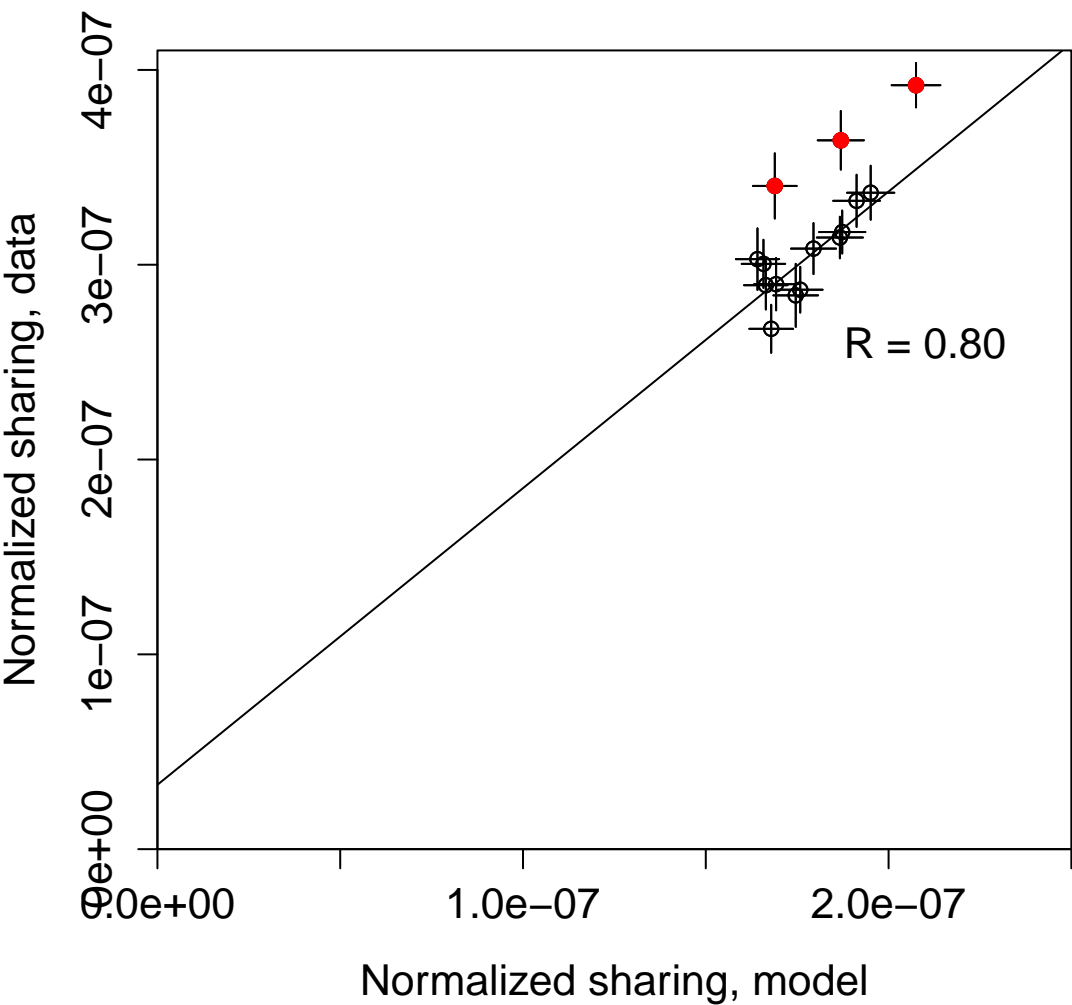

Supplement: S2 Fig — To be able to compare datasets of different sizes, the sharing number was normalized by the product of the two cloneset sizes. The outlying three red circles represent the twin pairs, while the black circles refer to pairs of unrelated individuals. Error bars show one standard deviation. The diagonal line is a linear fit for unrelated individuals, of slope 1.7. (PDF) [file pcbi.1005572.s003.pdf]

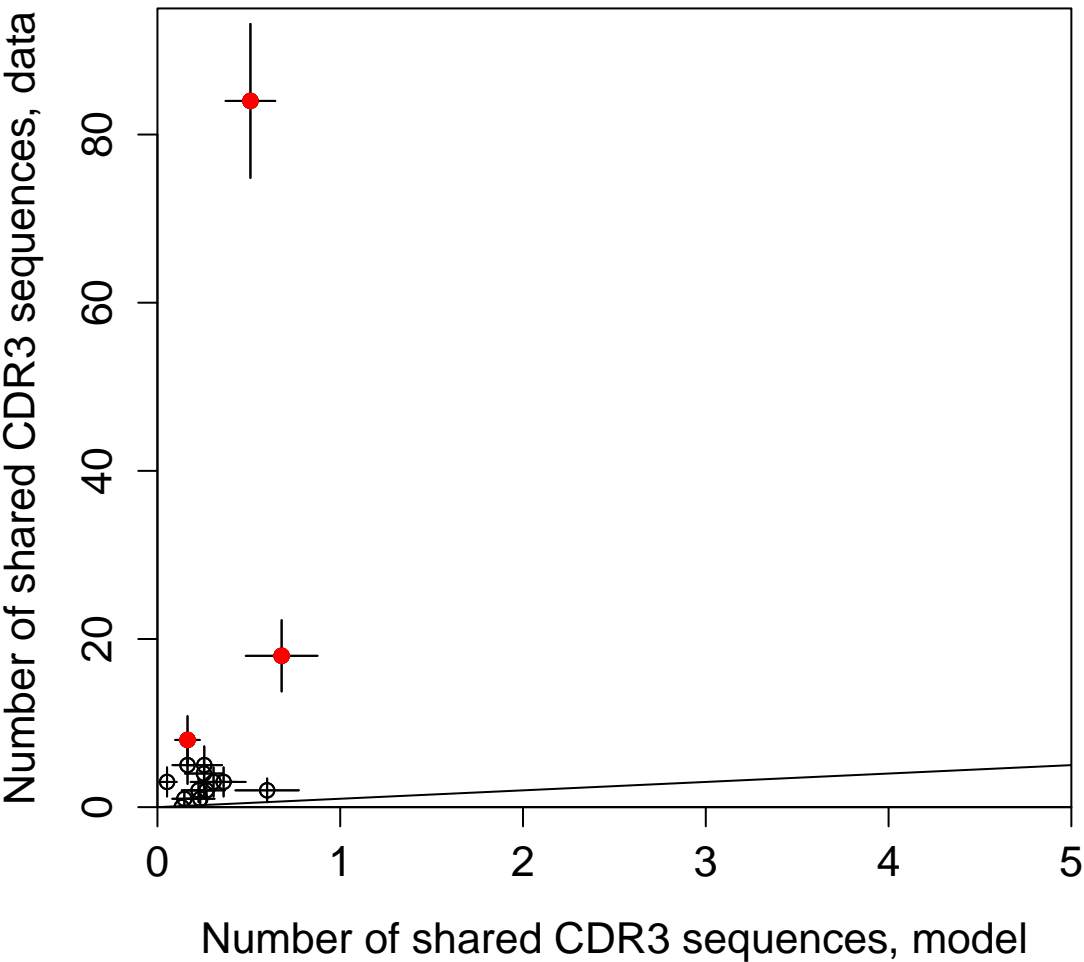

Supplement: S3 Fig — The three outlying red circles represent the twin pairs, while the black circles refer to pairs of unrelated individuals. Error bars show one standard deviation. (PDF) [file pcbi.1005572.s004.pdf]

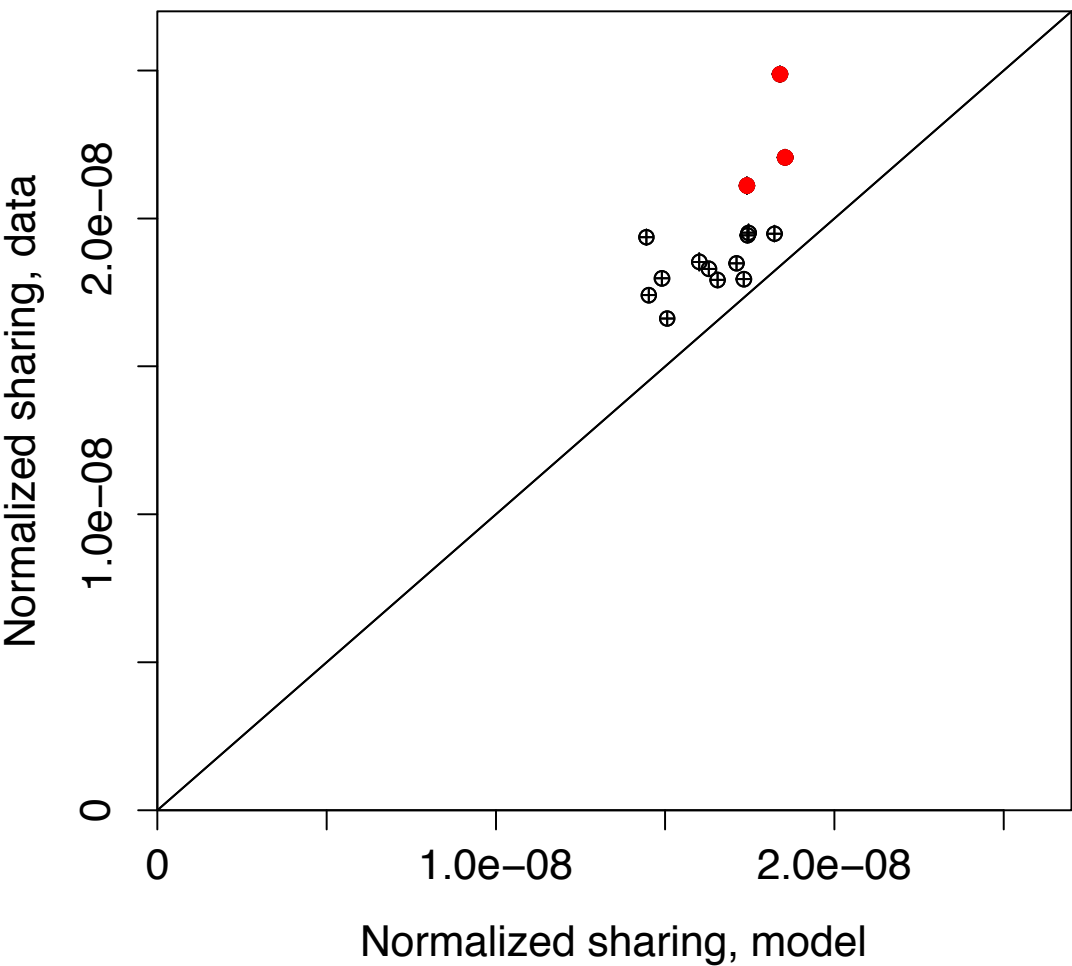

Supplement: S4 Fig — To be able to compare datasets of different sizes, the sharing number was normalized by the product of the two cloneset sizes. The three outlying red circles represent the twin pairs, while the black circles refer to pairs of unrelated individuals. Diagonal is equality line. Error bars show one standard deviation. (PDF) [file pcbi.1005572.s005.pdf]

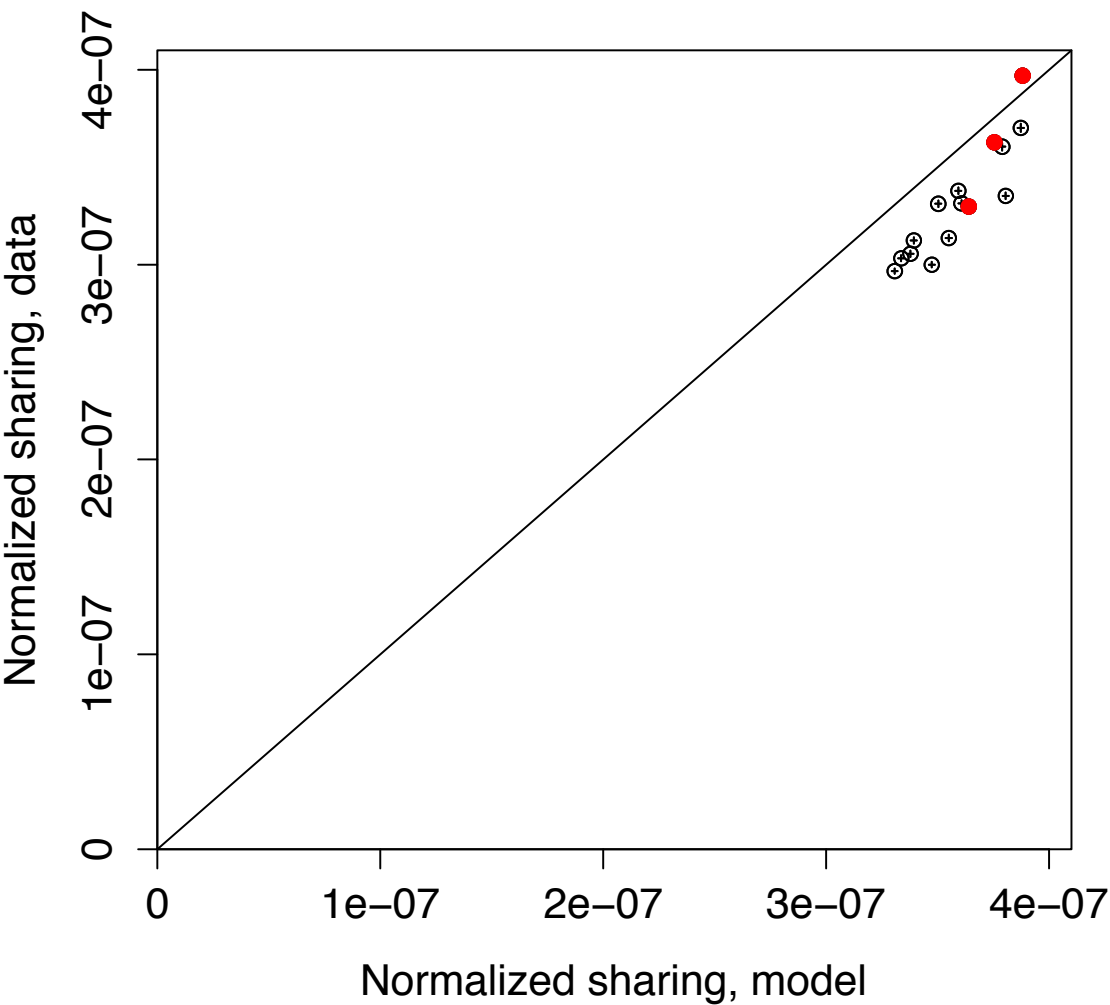

Supplement: S5 Fig — To be able to compare datasets of different sizes, the sharing number was normalized by the product of the two cloneset sizes. The three red circles represent the twin pairs, while the black circles refer to pairs of unrelated individuals. Diagonal is equality line. (PDF) [file pcbi.1005572.s006.pdf]

Mean number of insertions in shared clonotypes

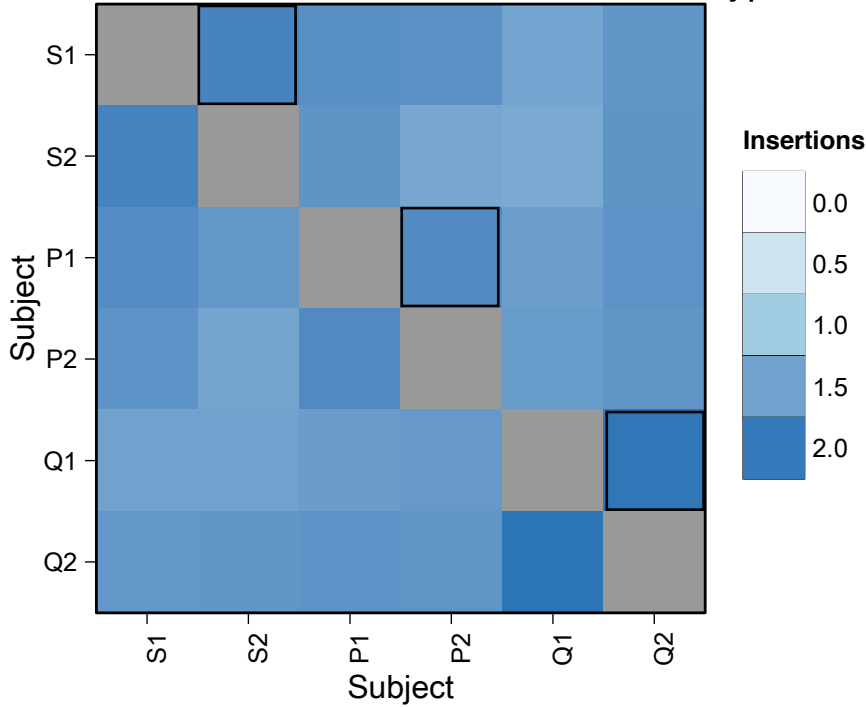

Supplement: S6 Fig — (PDF) [file pcbi.1005572.s007.pdf]

Mean number of insertions in shared clonotypes

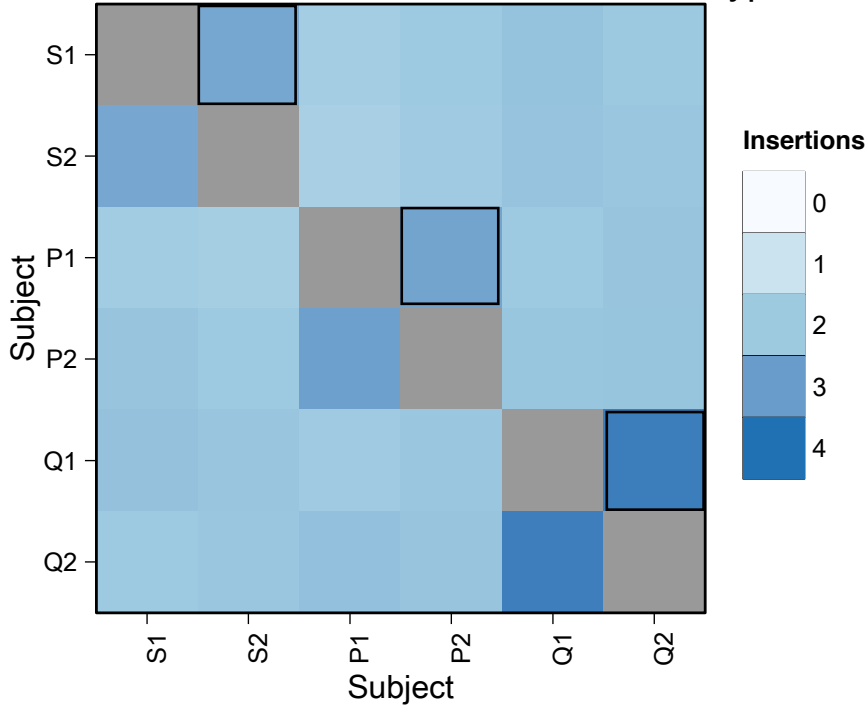

Supplement: S7 Fig — (PDF) [file pcbi.1005572.s008.pdf]

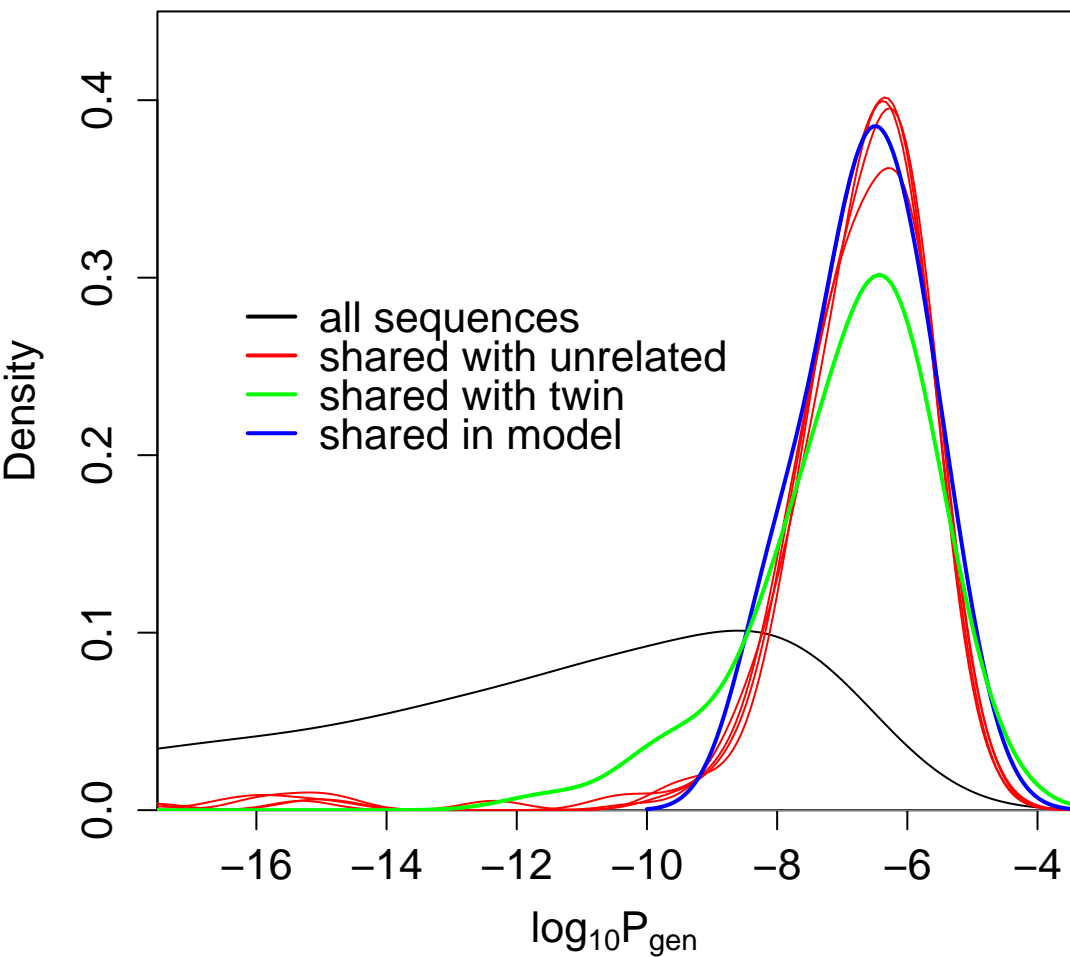

Supplement: S8 Fig — Distribution of Pgen—the probability that a sequence is generated by the VJ recombination process—for shared out-of-frame TCR alpha clonotypes between individual A1 from [8] and the other five individuals. While the distribution of shared sequences between unrelated individuals (red curves) is well explained by coincidental convergent recombination as predicted by our stochastic model (blue curve), sequences shared between two twins (green curve) have an excess of low probability sequences: 68 sequences with log10 Pgen < −10. For comparison the distribution of Pgen in regular (not necessarily shared) sequences is shown in black. (PDF) [file pcbi.1005572.s009.pdf]

Data normalized sharing

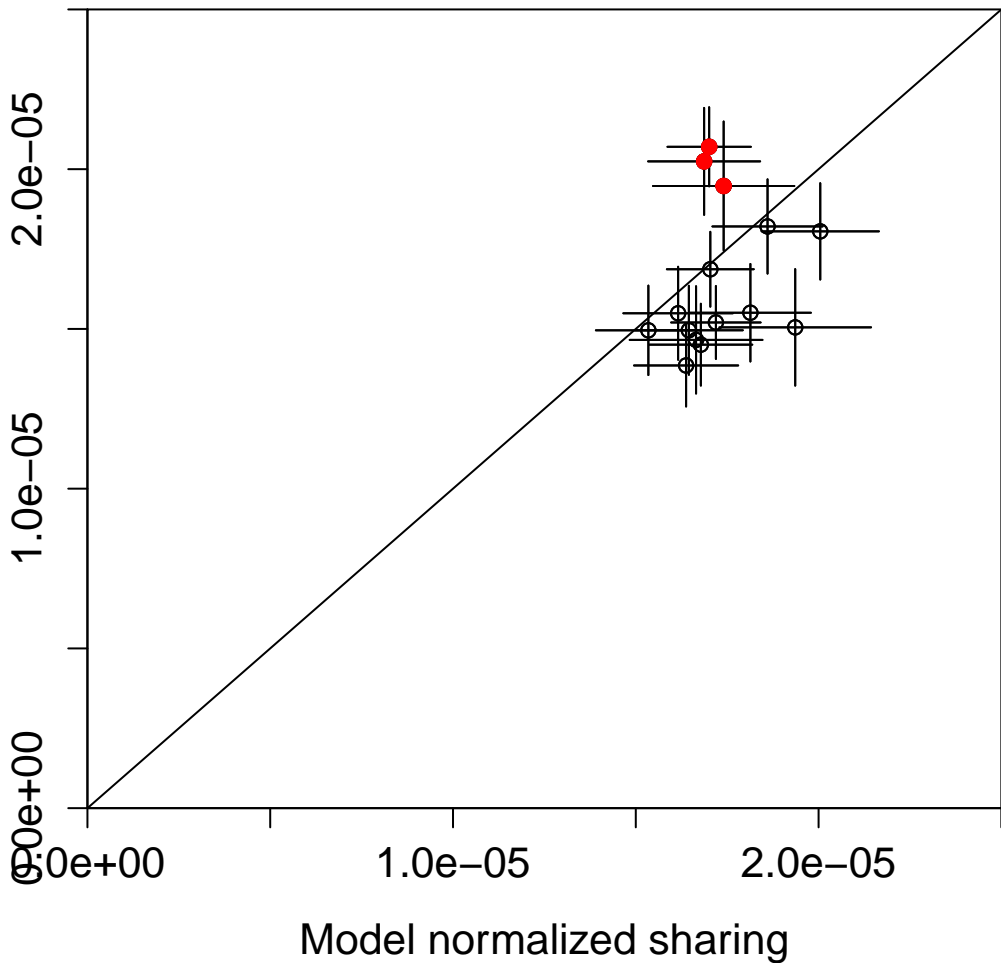

Supplement: S9 Fig — Number of shared out-frame alpha zero insertion TCR CDR3 clonotypes reported between all 15 pairs of 6 donors consisting of 3 twin pairs (ordinate) compared to the model prediction (abscissa). The three red circles represent the twin pairs, while the black circles refer to pairs of unrelated individuals. Diagonal is equality line. Error bars show one standard deviation. (PDF) [file pcbi.1005572.s010.pdf]

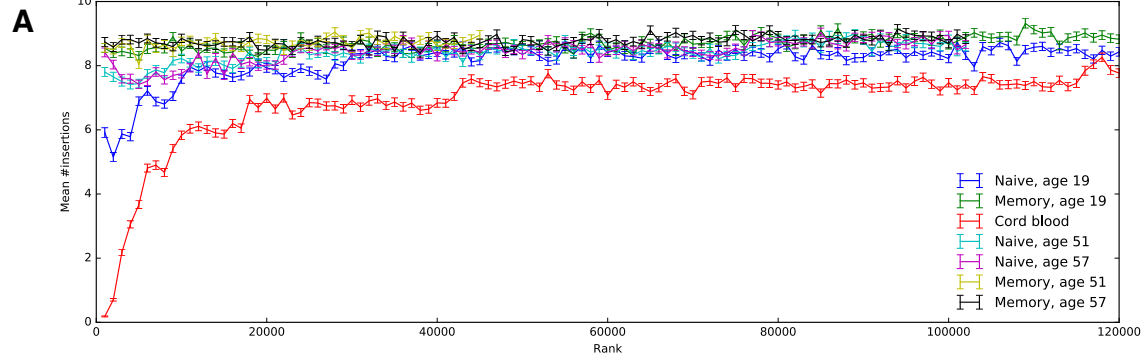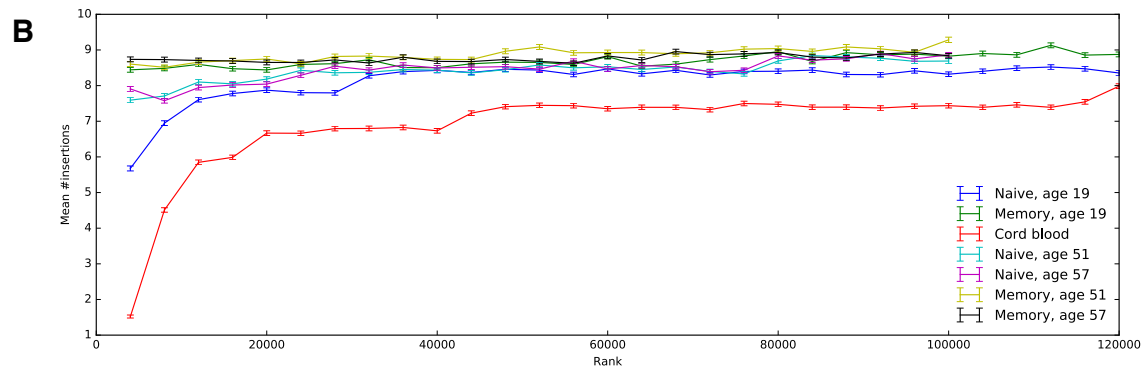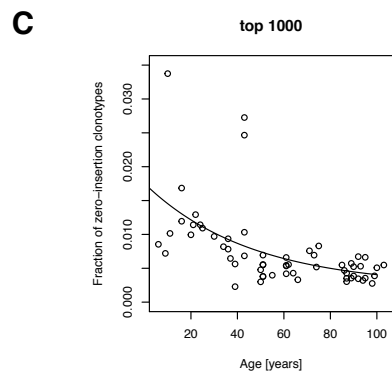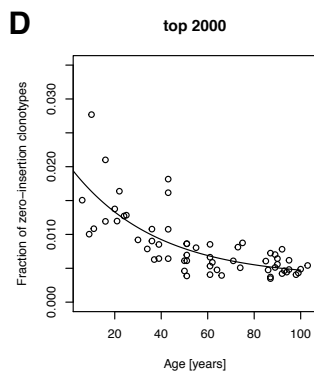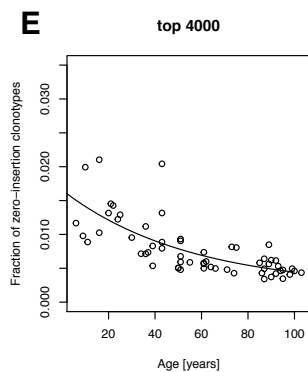

Supplement: S10 Fig — Mean numbers of insertions were obtained by analysing subsequent groups of 1000 (A) and 4000 (B) sequences of decreasing abundances, as in Fig 3A from the main text. (C,D,E) are results for ageing datasets reproduced for the top 1000, 2000 and 4000 clonotypes. Solid lines are independent fits to exponential decays (see main text Methods). Decay rate parameters for top 1000 and top 4000 clones are 0.0218 yr−1 and 0.0184 yr−1 respectively, within one standard error of the estimate for the top 2000 clones, 0.0272 ± 0.0091 yr−1. (PDF) [file pcbi.1005572.s011.pdf]

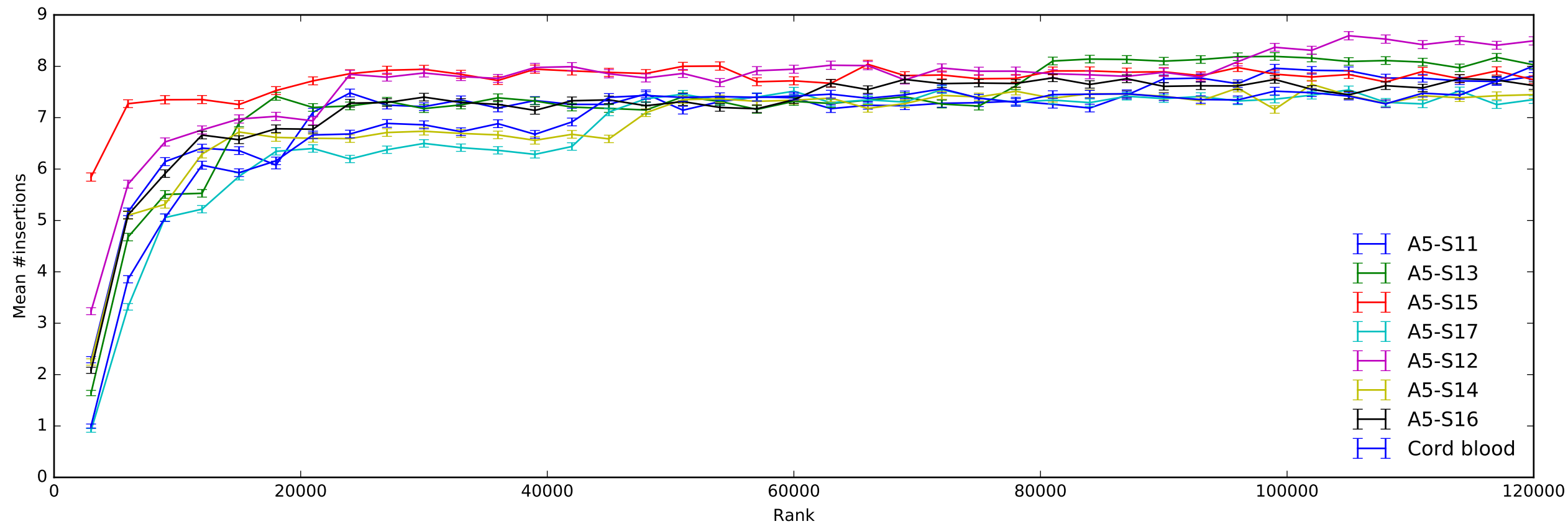

Supplement: S11 Fig — Mean numbers of insertions were obtained by analysing groups of 3000 sequences of decreasing abundances as in Fig 3A, for 7 independent published cord blood samples from [24]. A similar decreasing trend is observed for all samples. (PDF) [file pcbi.1005572.s012.pdf]

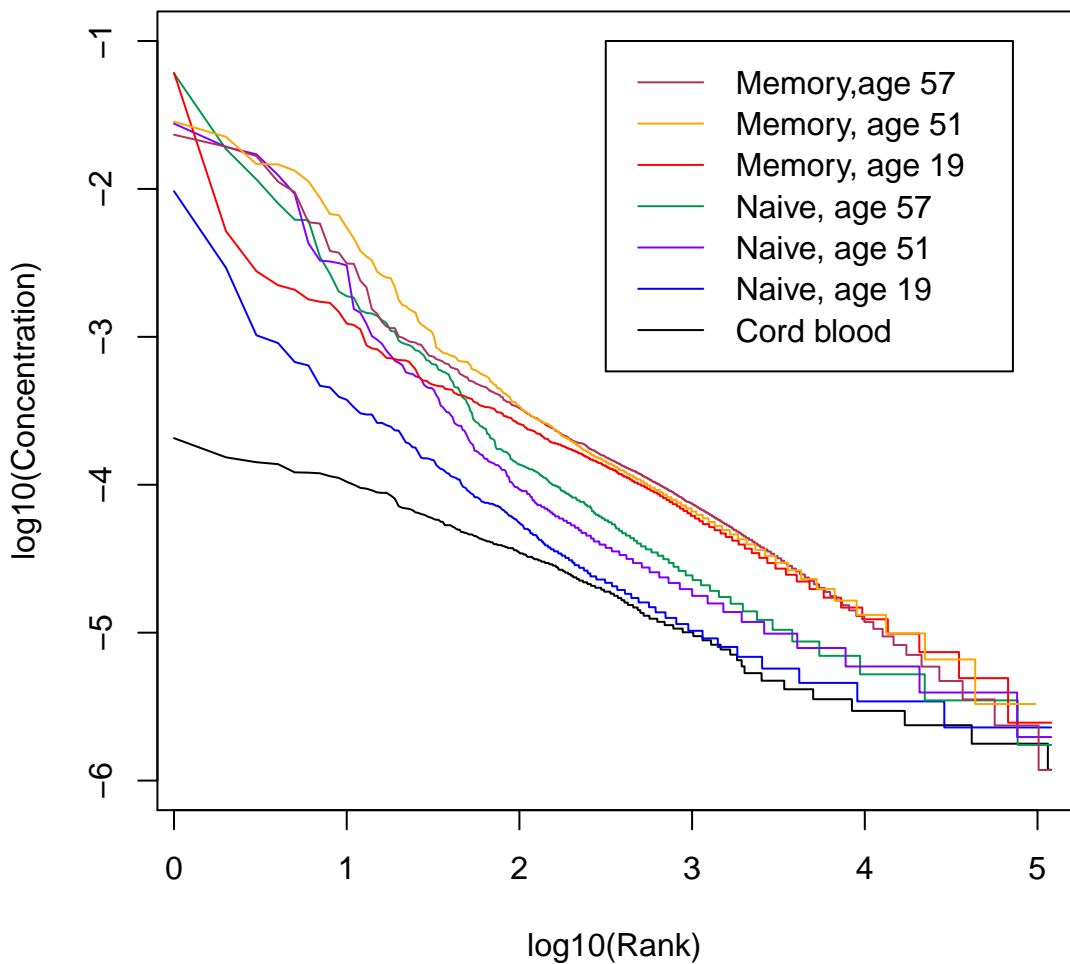

Supplement: S12 Fig — Here we show the dependence of the clone abundance on its abundance rank in samples from Fig 3A. Memory clones are typically larger than the naive and cord blood clones of same rank, possibly due to the history of clonal expansions. (PDF) [file pcbi.1005572.s013.pdf]
